# A drug-free nanozyme for mitigating oxidative stress and inflammatory bowel disease

**DOI:** 10.1186/s12951-022-01319-7

**Published:** 2022-03-04

**Authors:** Feng Zeng, Yahong Shi, Chunni Wu, Jianming Liang, Qixin Zhong, Karen Briley, Bin Xu, Yongzhuo Huang, Manmei Long, Cong Wang, Jian Chen, Yonghua Tang, Xinying Li, Mengda Jiang, Luting Wang, Qin Xu, Liu Yang, Peng Chen, Shengzhong Duan, Jingyuan Xie, Cong Li, Yingwei Wu

**Affiliations:** 1 Artemisinin Research Center, Institute of Science and Technology, The First Affiliated Hospital, The First Clinical Medical School, Lingnan Medical Research Center, Guangzhou University of Chinese Medicine, Guangzhou, 510450 China; 2 Department of Radiology, Shanghai Ninth People’s Hospital, Shanghai Jiao Tong University School of Medicine, Shanghai, 200011 China; 3 Key Laboratory of Smart Drug Deliver, Ministry of Education, School of Pharmacy, Fudan University, Shanghai, 201213 China; 4 Department of Nephrology, Institute of Nephrology, Shanghai Ruijin Hospital, Shanghai Jiao Tong University School of Medicine, Shanghai, 200020 China; 5 Shanghai Institute of Materia Medica, Chinese Academy of Sciences, Shanghai, 201203 China; 6 Zhongshan Institute for Drug Discovery and Development, Chinese Academy of Sciences, Zhongshan, 528437 China; 7 School of Advanced Study, Institute of Natural Medicine and Health Product, Taizhou University, Taizhou, 318000 China; 8 China Academy for Engineering and Technology, Fudan University, Shanghai, 200433 China; 9 Laboratory of Oral Microbiota and Systemic Diseases, Shanghai Ninth People’s Hospital, College of Stomatology, Shanghai Jiao Tong University School of Medicine, Shanghai, 200125 China; 10 National Clinical Research Center for Oral Diseases, Shanghai Key Laboratory of Stomatology and Shanghai Research Institute of Stomatology, Shanghai, 200011 China; 11 Department of Pathology, Shanghai Ninth People’s Hospital, Shanghai Jiao Tong University School of Medicine, Shanghai, 200011 China; 12 Department of Molecular Diagnostics, The Core Laboratory in Medical Center of Clinical Research, Department of Endocrinology, State Key Laboratory of Medical Genomics, Shanghai Ninth People’s Hospital, Shanghai Jiao Tong University School of Medicine, Shanghai, 200011 China; 13 Radiology Department, Ruijin Hospital, Shanghai Jiao Tong University School of Medicine, Shanghai, 200020 China; 14 Department of Gastroenterology, Ruijin Hospital, Shanghai Jiao Tong University School of Medicine, Shanghai, 200020 China; 15 Department of Cardiovascular, Shenzhen Hospital of Guangzhou University of Chinese Medicine, Shenzhen, 518034 China; 16 Invicro, A Konica Minolta Company, Boston, MA 02210 USA

**Keywords:** Ceria nanoparticles, Reactive oxygen species, Inflammatory bowel disease, Macrophages, Proinflammatory microenvironment

## Abstract

Inflammatory bowel disease (IBD) is an incurable disease of the gastrointestinal tract with a lack of effective therapeutic strategies. The proinflammatory microenvironment plays a significant role in both amplifying and sustaining inflammation during IBD progression. Herein, biocompatible drug-free ceria nanoparticles (CeNP-PEG) with regenerable scavenging activities against multiple reactive oxygen species (ROS) were developed. CeNP-PEG exerted therapeutic effect in dextran sulfate sodium (DSS)-induced colitis murine model, evidenced by corrected the disease activity index, restrained colon length shortening, improved intestinal permeability and restored the colonic epithelium disruption. CeNP-PEG ameliorated the proinflammatory microenvironment by persistently scavenging ROS, down-regulating the levels of multiple proinflammatory cytokines, restraining the proinflammatory profile of macrophages and Th1/Th17 response. The underlying mechanism may involve restraining the co-activation of NF-κB and JAK2/STAT3 pathways. In summary, this work demonstrates an effective strategy for IBD treatment by ameliorating the self-perpetuating proinflammatory microenvironment, which offers a new avenue in the treatment of inflammation-related diseases.

**Graphical Abstract:**

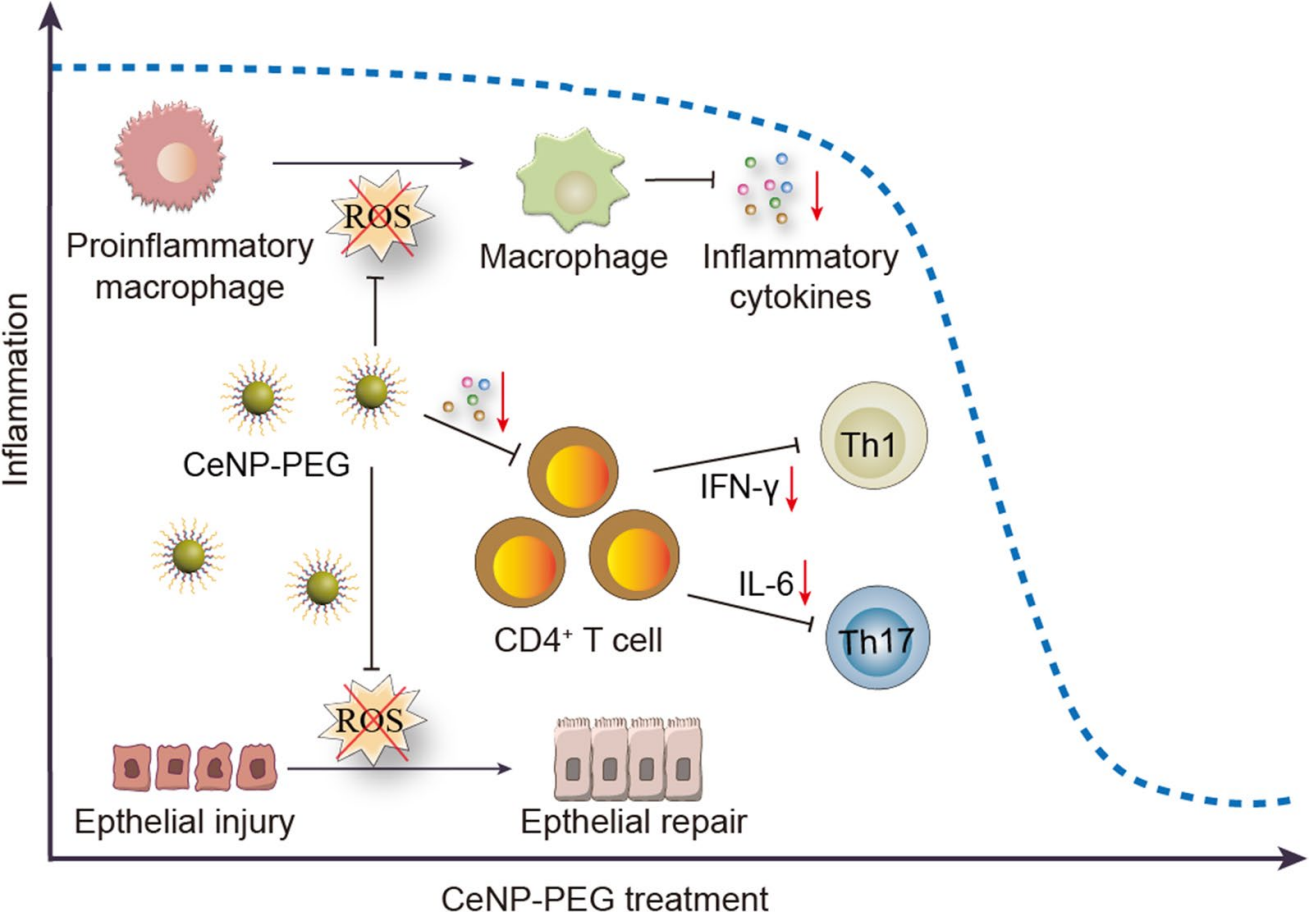

**Supplementary Information:**

The online version contains supplementary material available at 10.1186/s12951-022-01319-7.

## Introduction

Inflammatory bowel disease (IBD), including ulcerative colitis (UC) and Crohn’s disease (CD), are characterized by immune-mediated refractory chronic inflammation in the gastrointestinal tract [1]. To date, the exact etiology of IBD is still incompletely understood; however, it is generally thought to be a result of an inappropriate immune response of the innate (such as macrophages) and adaptative (such as T cells) immune systems [2, 3]. Although biological therapies have significantly improved patient management, only 25% of patients with IBD achieve sustained disease remission after administration of current anti-TNF biologics. Another 30% of the IBD patient population still requires total colectomy, highlighting the complex pathogenesis and current limitations of available medical treatments of IBD [4]. Thus, innovative treatment strategies to improve therapeutic efficacy are greatly needed.

Accumulating evidence strongly suggests that the proinflammatory microenvironment in the intestine plays a significant role in both amplifying and sustaining inflammation during IBD progression [5–7]. The continued increase of reactive oxygen species (ROS) is one of the key drivers for maintaining the proinflammatory microenvironment within the intestines [8, 9]. For example, excessive ROS generated from inflammation are critical for the proinflammatory activation of macrophages, which causes the continued release of proinflammatory mediators, such as IL-1β, IL-6, TNF-α, IFN-γ and continued release of ROS [10–12]. In addition, several proinflammatory cytokines, such as IL-6 and IFN-γ, also induce T-cell accumulation and apoptosis resistance that further promotes chronic inflammation [13]. Thus, the interplays triggered and maintained by the overproduced ROS within the proinflammatory microenvironment results in a self-perpetuation vicious cycle that greatly contributes the pathogenesis of IBD [10, 14]. Therefore, we posit that disrupting the self-perpetuation vicious cycle by eradicating ROS may be of great potential to ameliorate the proinflammatory microenvironment and promote the mucosal healing in IBD.

Ceria nanoparticles (CeNP) are robust nanozymes that can scavenge multiple ROS and their capability of anti-oxidative and anti-inflammatory effects have been demonstrated in both cell and animal models due to the coexistence of reversible Ce^3+^ (reduced) and Ce^4+^ (oxidized) sites on the particle surface [15–19]. The Ce^4+^ sites are able to eradicate hydrogen peroxide (H_2_O_2_) via catalase (CAT)-mimetic activity, while Ce^3+^ sites are responsible for eliminating hydroxyl radicals (•OH) and superoxide anions (O_2_^•−^) via redox reactions or superoxide dismutase (SOD)-mimetic activity, respectively [18, 20]. Other stoichiometric antioxidants are consumed as they scavenged ROS and facilitating catalytic activity against just one specific ROS subtype [19]. The repeated administration of large amounts of active agent may increases the potential toxicity to biosystem [19]. Moreover, CeNP showed additional advantages, such as high efficiency, high versatility, high stable, low costs, and minimal immunogenicity [21]. We previously demonstrated that CeNP exhibited neuroprotective effects by suppressing the proinflammatory responses of activated microglia [16]. This study clearly suggested that CeNP can alleviate the inflammation via suppressing the proinflammatory functions of macrophages, which is consistent with the results focusing on other inflammatory related diseases such as rheumatoid arthritis (RA) [22]. However, there are many complex examples such as myeloid cells co-existence with a mixed phenotype, which result from the balance of regulators present in the tissue microenvironment [10]. Therefore, the unanswered question whether CeNP can ameliorate the proinflammatory microenvironment is highly of importance to be addressed.

Herein, we demonstrate the correlation between proinflammatory macrophages and disease severity in UC patients based on computed tomographic enterography (CTE) data, endoscopic images, biochemical measurements, and intestinal tissue biopsies. Polyethylene glycol (PEG)-capped ultrafine CeNP (CeNP-PEG) with excellent ROS scavenging activity were prepared. To identify the ability of CeNP-PEG in ameliorating the proinflammatory microenvironment of IBD, the ROS levels, barrier function of the intestinal epithelium, proinflammatory cytokine levels, intestinal macrophage function, and the Th1/Th17 response were determined by using dextran sulfate sodium (DSS)-induced colitis murine models. In vivo CTE and [^18^F]-DPA-714 dynamic PET/CT imaging were performed to allow for the evaluation of both active inflammation and active macrophage density as a function of CeNP-PEG treatment. The possible signaling pathways were also investigated to identify the potential mechanism of action associated with CeNP-PEG treatment for IBD. These findings collectively document that CeNP-PEG treatment disrupts the circuitry of the proinflammatory microenvironment in IBD, which offers a new therapeutic concept for any other chronic inflammatory conditions.

## Results

### Upregulation of proinflammatory macrophages and cytokines in intestinal tissue of UC patients

**Fig. 1:**
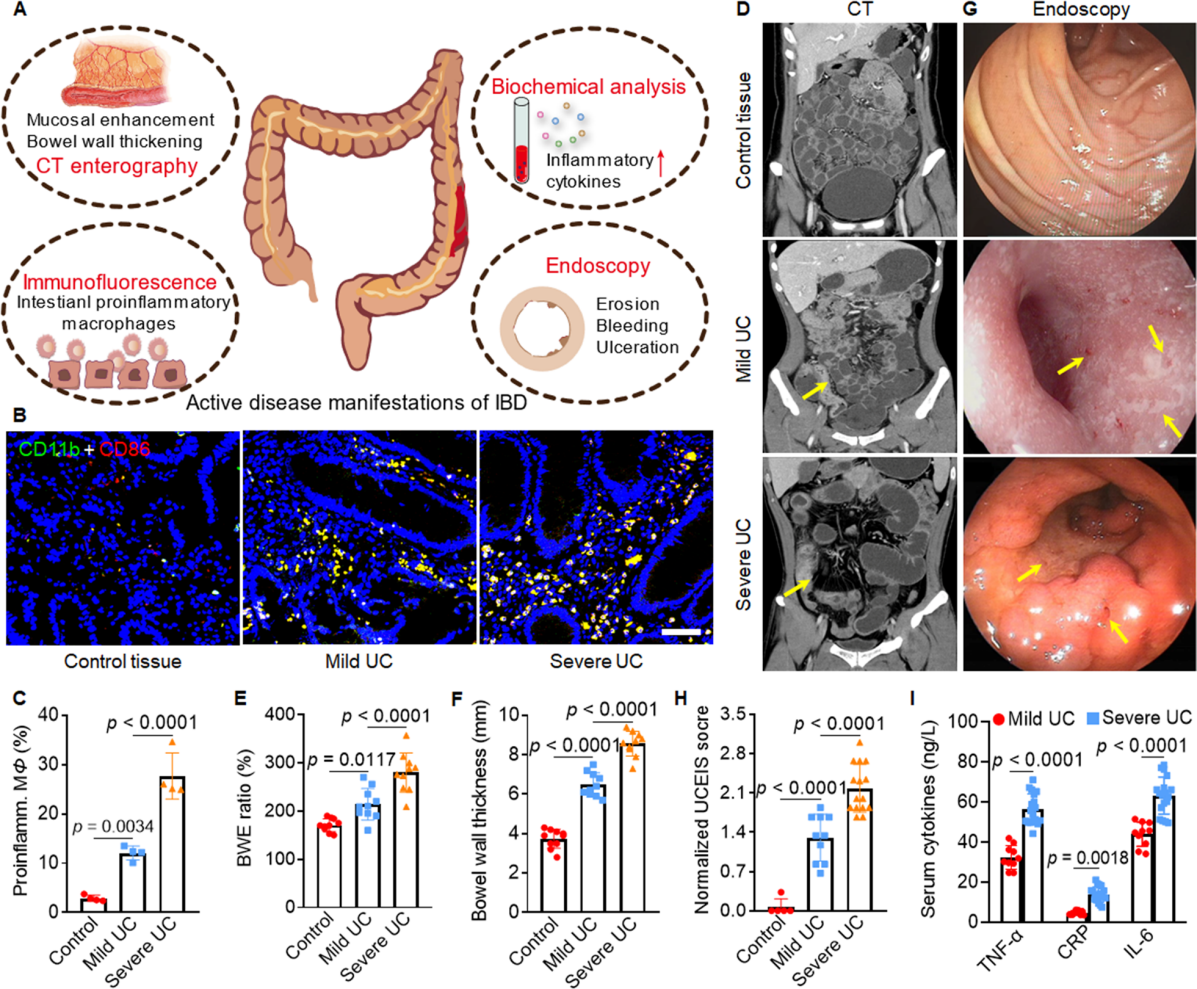
Disease severity correlated with the proinflammatory macrophage percentage and proinflammatory cytokine levels in UC patients. **A** Schematic diagram of active disease manifestations of IBD. **B** Representative immunofluorescence images of colon specimens from UC patients costained with the proinflammatory macrophage phenotype biomarkers CD11b (green color) and CD86 (red color). The nuclei were stained blue with 4,6-diamidino-2-phenylindole (DAPI). Scale bar = 50 μm. **C** The percentages of proinflammatory macrophages in the colon specimens of healthy controls or patients with UC (n = 4). **D** Representative coronal computed tomographic enterography (CTE) images showed abnormal CT manifestations in patients with ulcerative colitis (UC). Yellow arrows indicate segmental bowel wall thickening with strong mucosal enhancement in both mild and severe UC. Measurements of bowel wall enhancement (the ratio of venous stage enhancement to plain scan) (**E**) and bowel wall thickness (**F**) in patients with UC (n = 10). **G** Representative endoscopic images of healthy controls and patients with mild or severe UC. Yellow arrows indicate mucosal erosion, ulceration, and reductions in the vascular pattern, which are symptoms of intestinal inflammation. **H** The endoscopic severity score was assessed by the normalized UC endoscopic index of severity (UCEIS) (healthy controls n = 5, mild UC patients n = 10, and serve UC patients n = 15). **I** The serum levels of proinflammatory cytokines in patients with mild or severe UC (mild UC patients n = 10 and severe UC patients n = 18). The data are presented as the mean ± SD. One-way ANOVA was used for statistical analysis. Proinflamm. M*Φ*: Proinflammatory macrophage

Clinical data of UC patients, including CTE images, endoscopic images, biochemical measurements, and immunofluorescence staining, were examined to assess the relationship between proinflammatory macrophages and disease severity (Fig. 1A). Immunofluorescence costaining with CD11b^+^ and CD86^+^ revealed that the percentage of proinflammatory macrophages in mild UC patients and severe UC patients was 12.1 and 27.7 times that of healthy controls, respectively (Fig. 1B and C). The CTE images revealed bowel wall enhancement ratios in healthy control, mild UC and severe UC patients of 170.9% ± 13.4%, 214.2% ± 32.6%, and 279.9% ± 40.7%, respectively. The corresponding bowel wall thickness was 3.7 ± 0.5 mm, 6.5 ± 0.6 mm, and 8.7 ± 0.6 mm, respectively, indicating higher severity in UC patients with stronger bowel wall enhancement and increased bowel wall thickness in CTE images (Fig. 1D–F). Endoscopic images showed that patients with mild UC had mucosal erosion, superficial ulcers, and a reduction in the vascular pattern. Patients with severe UC exhibited colonic ulceration, spontaneous bleeding, and an absent in vascular pattern (Fig. 1G). We used the normalized UC endoscopic index of severity (UCEIS) score to evaluate disease severity and found that the scores correlated with disease severity; the mean normalized UCEIS score was 0.1 ± 0.1 in healthy controls, 1.3 ± 0.4 in mild UC patients and 2.2 ± 0.4 in severe patients (Fig. 1H). Biochemical analysis exhibited that the serum levels of the proinflammation cytokines TNF-α, CRP, and IL-6 were 1.7, 2.9, and 1.4 times higher in severe UC patients, relative to patients with mild UC (Fig. 1I, Additional file 1: Table S1). The clinical data indicated a correlation between disease severity and proinflammatory macrophages and cytokine levels in the intestinal tissues of UC patients. This data strongly suggests the important role of proinflammatory macrophages in IBD progression.

### Synthesis and characterization of ceria nanoparticles

**Fig. 2:**
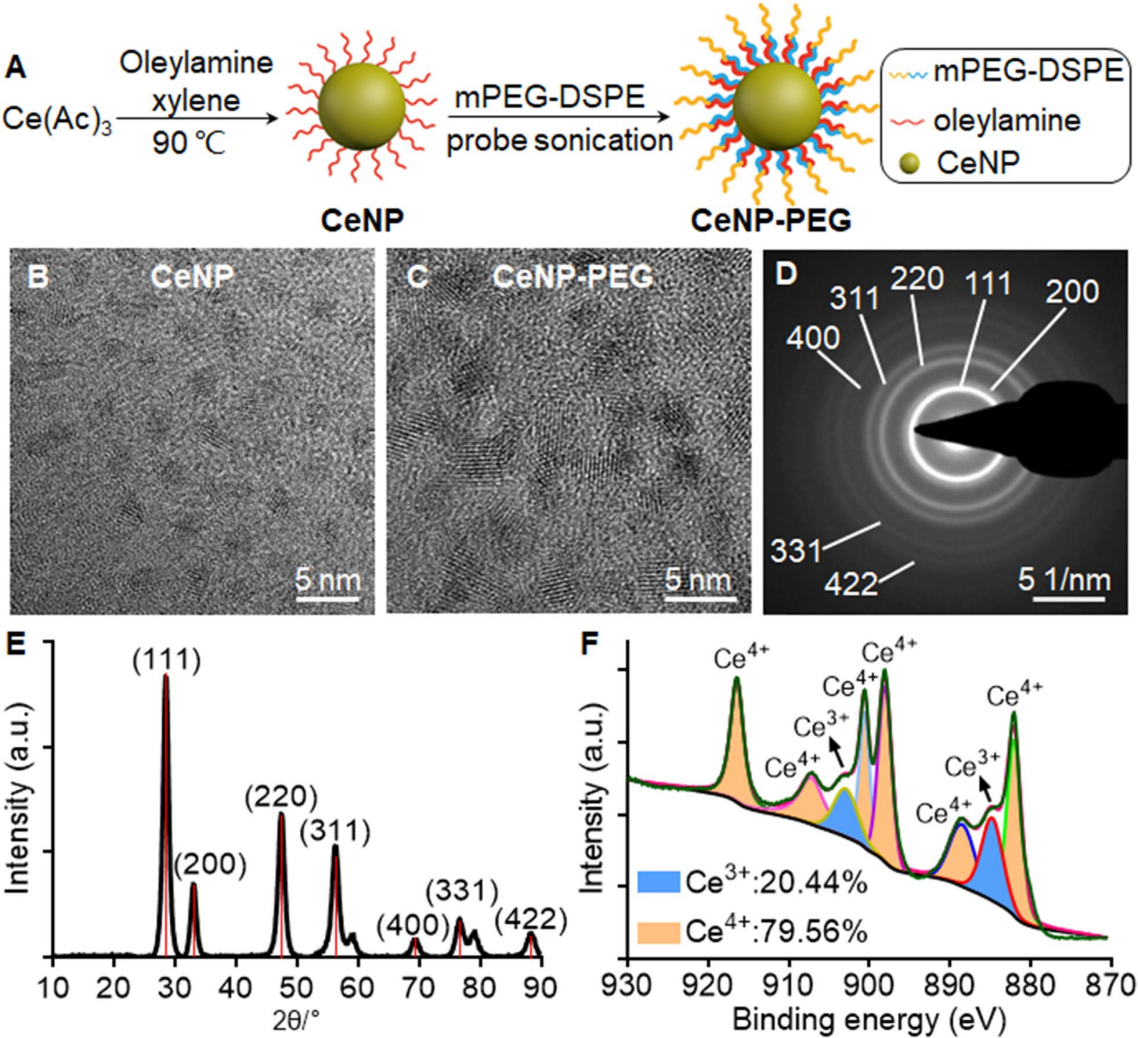
Synthesis and characterization of ceria nanoparticle. **A** The synthetic procedure of CeNP and CeNP-PEG. Representative high-resolution TEM image of CeNP (**B**) and CeNP-PEG (**C**). The SAED (**D**) and XRD (**E**) patterns of CeNP indicated a pure and typical cubic fluorite structure. **F** XPS spectrum of Ce3d revealed the valence state and the corresponding binding energy peaks of Ce (III) (884.8 and 902.9 eV) and Ce (IV) (882.1, 888.5, 898.1, 900.6, 907.2, and 916.4 eV). a.u.: arbitrary units

The hydrophobic ultrasmall nanozyme CeNP was synthesized based on a nonhydrolytic sol–gel reaction with slight modifications [20] and was transferred to a hydrophilic phase with a PEG coating to improve biocompatibility for further biomedical applications (Fig. 2A) [16]. Transmission electron microscopy (TEM) images of the CeNP revealed a uniform, discrete, and highly crystalline spherical morphology with a size of about 2.5 nm (Fig. 2B and C, Additional file 1: Fig. S1). Energy-dispersive spectroscopy (EDS) showed that the CeNP was composed of Ce and O (Cu and C come from the carbon film) (Additional file 1: Fig. S2). The cubic fluorite structure of CeNP was confirmed with selected-area electron diffraction (SAED) and X-ray diffraction (XRD) analyses (Fig. 2D and E). X-ray photoelectron spectroscopy (XPS) revealed a mixed-valence state of Ce^3+^ (peaks at 884.8 and 902.9 eV) and Ce^4+^ (882.1, 888.5, 898.1, 900.6, 907.2, and 916.4 eV) and with appoximately 20.44% Ce^3+^ on the nanozyme surface (Fig. 2F) [18]. The hydrodynamic diameter of the hydrophilic nanozyme CeNP-PEG was determined to be 10.15 ± 0.69 nm with a polymer dispersity index (PDI) of 0.35 ± 0.01, and the ζ-potential value was − 7.36 ± 0.05 mV (Additional file 1: Fig. S3).

### CeNP-PEG with high cellular uptake efficiency and biocompatibility

Fluorescence-conjugated CeNP-PEG were used to investigate the cellular uptake of bone marrow-derived macrophages (BMDMs). The results showed that cellular uptake was obvious as early as 15 min after the addition of 1.0 μg/mL CeNP-PEG or as little as 125 ng/mL CeNP-PEG within a 2 h incubation time and increased consistently with time or CeNP-PEG concentration, indicating that the cellular uptake of CeNP-PEG was concentration- and time-dependent (Additional file 1: Figs. S4 and S5). Cell Counting Kit-8 (CCK-8) assays were used to study the cytotoxicity of CeNP-PEG. CeNP-PEG did not show any cytotoxicity at concentrations up to 10 μg/mL and the viability of BMDMs remained above 80% when the concentration of CeNP-PEG was as high as 20 μg/mL (Additional file 1: Fig. S6), indicating their excellent biocompatibility.

### Multiple ROS scavenging capability of CeNP-PEG

**Fig. 3:**
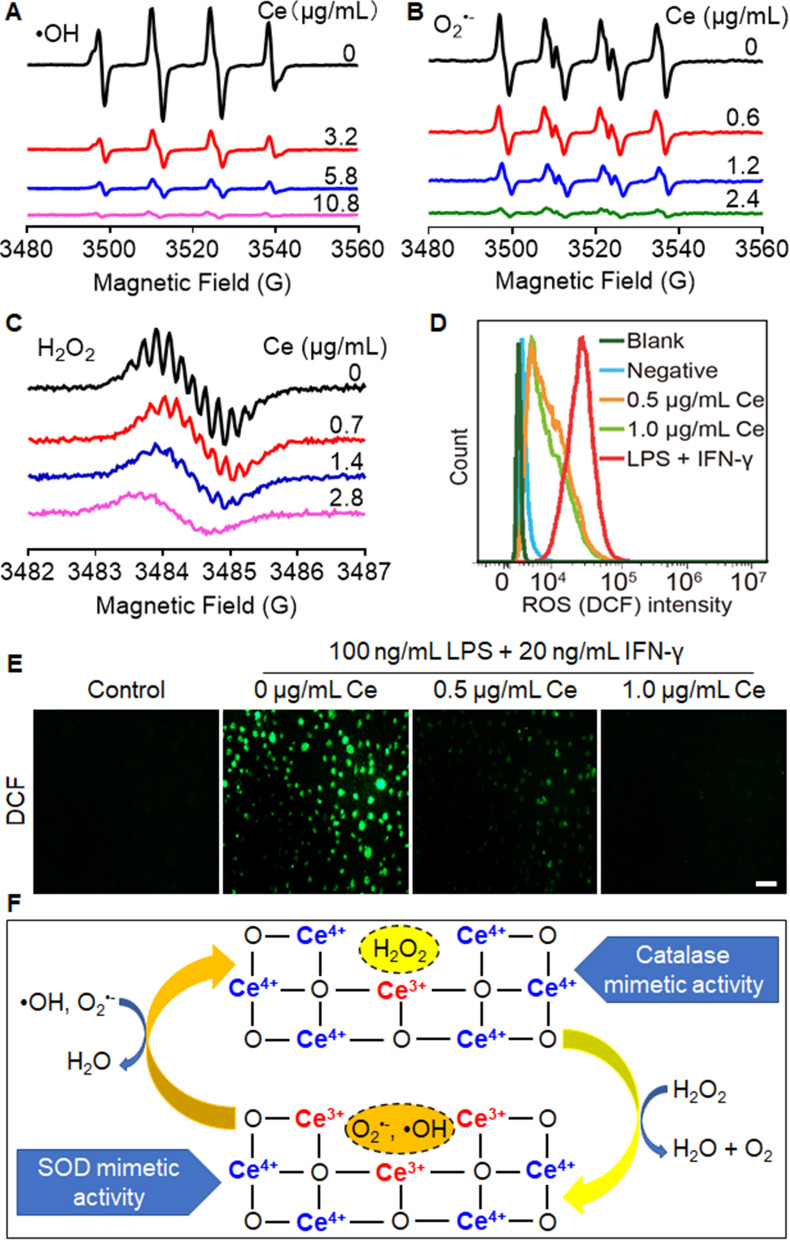
CeNP-PEG exhibited high antioxidant activities against multiple ROS. ESR spectra showing concentration-dependent scavenging activities of CeNP-PEG to ROS, including •OH (**A**), O_2_^•−^ (**B**) and H_2_O_2_ (**C**). **D** Intracellular ROS levels in BMDMs was determined by flow cytometry after the treatment with complete medium, 100 ng/mL LPS + 20 ng/mL IFN-γ in the presence or absence of CeNP-PEG (0.5 or 1.0 μg/mL). **E** Representative fluorescence microscopy images of BMDMs isolated from mice after the indicated treatments. Scale bar = 100 μm. **F** A proposed schematic illustration of the antioxidant mechanism of CeNP-PEG against multiple ROS via reversible valence state switching between Ce^3+^ and Ce^4+^. BMDMs: Bone marrow-derived macrophages. LPS: Lipopolysaccharide. DCF: 2’, 7’-Dichlorodihydrofluorescein

To demonstrate the anti-ROS cascade reaction effect of CeNP-PEG, the •OH elimination activity and SOD- and CAT-mimetic activities of CeNP-PEG were investigated by electron spin resonance (ESR) spectroscopy. •OH and O_2_^•−^ were stabilized by the spin trapping agent 5,5-dimethyl-1-pyrroline N-oxide (DMPO) by forming the spin adduct DMPO/•OH and DMPO/•OOH, respectively, and the oxygen-sensitive spin-label probe 3-carbamoyl-2,5-dihydro-2,2,5,5-tetramethyl-1H-pyrrol-1-yloxyl (CTPO) was used to measure O_2_ generation from the decomposition of H_2_O_2_ by CeNP-PEG. Notably, the ESR spectra of DMPO/•OH, DMPO/•OOH, and the superhyperfine structure of CTPO sharply decreased after the addition of various concentrations of CeNP-PEG, verifying the capability of CeNP-PEG to scavenge multiple ROS (Fig. 3A–C). BMDMs were treated with 100 ng/mL LPS + 20 ng/mL IFN-γ to induce proinflammatory macrophages and produce a large amount of ROS. Flow cytometry showed that while the median ROS fluorescence intensity increased 9.9 times after LPS + IFN-γ stimulation, this level decreased by 75.4% and 79.5% after pretreatment with 0.5 and 1.0 μg/mL CeNP-PEG (Fig. 3D). Similarly, fluorescence microscopy showed that ROS fluorescence intensity in BMDMs increased markedly after treatment with LPS + IFN-γ and decreased by 64.3% and 90.3% after pretreatment with 0.5 and 1.0 μg/mL CeNP-PEG (Fig. 3E, Additional file 1: Fig. S7), suggesting the potent antioxidant effect of CeNP-PEG in biological environments. Figure 3F shows the proposed schematic illustration of the CAT-mimetic, SOD-mimetic, and hydroxyl radical scavenging activities of CeNP-PEG and reversible valence state switching between Ce^3+^ and Ce^4+^.

### CeNP-PEG suppresses proinflammatory cytokine production and proinflammatory macrophages

**Fig. 4:**
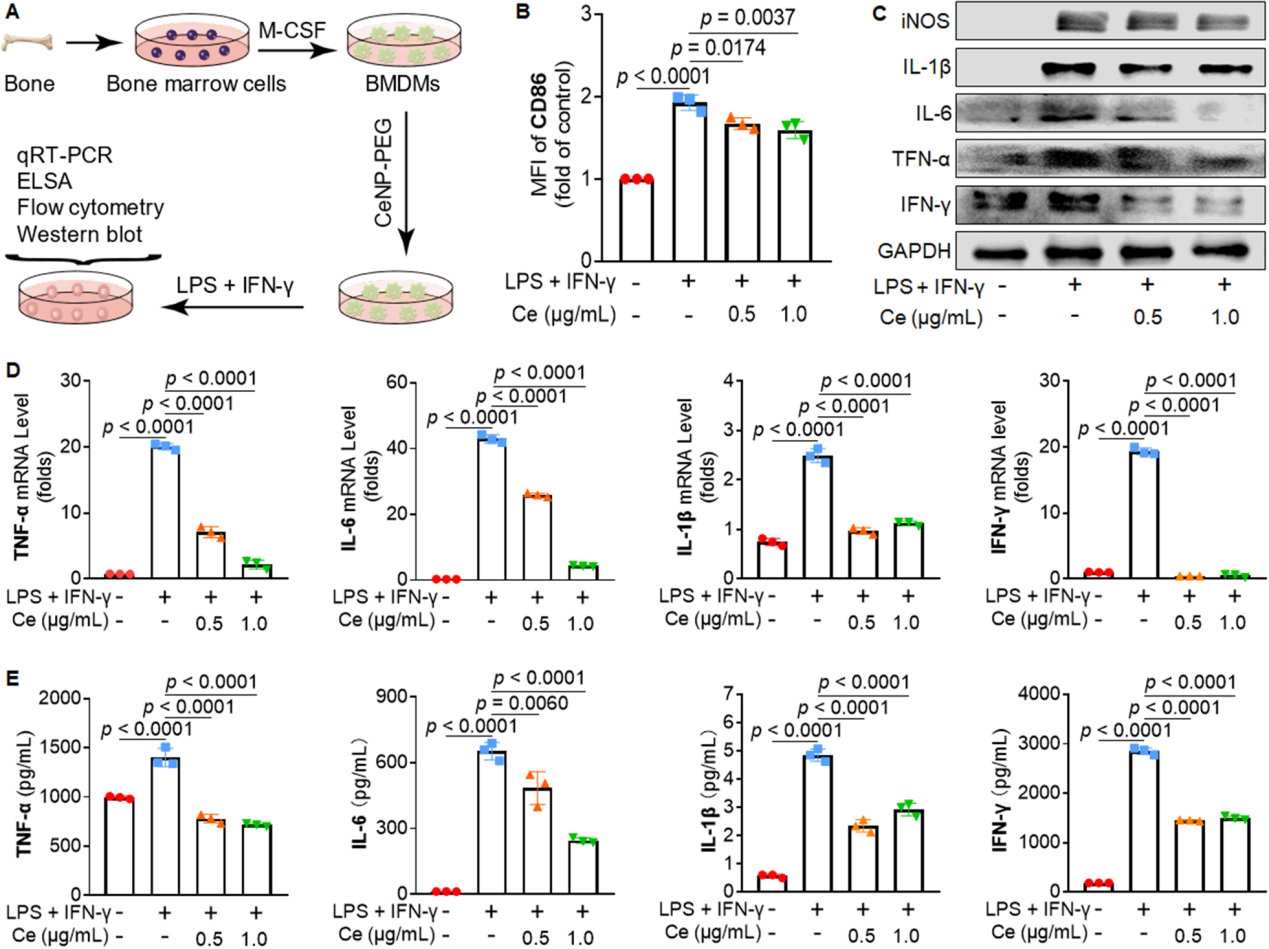
CeNP-PEG inhibited the proinflammatory profile of macrophages. **A** Schematic diagram of the experimental setup to evaluate the effect of CeNP-PEG decreased the proinflammatory profile of BMDMs. **B** CD86 expression levels in BMDMs treated with different concentrations of CeNP-PEG. **C** Western blotting analysis of proinflammatory phenotype biomarkers including iNOS, IL-1β, IL-6, TNF-α, and INF-γ in BMDMs. **D** The mRNA levels of TNF-α, IL-6, IL-1β and IFN-γ in BMDMs after treatment with complete medium, 100 ng/mL LPS + 20 ng/mL IFN-γ in the presence or absence of CeNP-PEG (0.5 or 1.0 μg/mL). **E** The concentrations of TNF-α, IL-6, IL-1β, and IFN-γ in BMDMs culture media after the above treatments. The data are presented as the mean ± SD (n = 3). One-way ANOVA was used for statistical analysis. MFI: mean fluorescence intensity

Proinflammatory macrophages are inflammatory immune cells that produce many proinflammatory cytokines and ROS and then trigger inflammatory responses [23]. BMDMs were used to determine whether CeNP-PEG can suppress the proinflammatory phenotype of macrophage and the production of proinflammatory cytokines (Fig. 4A). Flow cytometry revealed that CD86, a surface marker for proinflammatory macrophages, was pronounced in LPS + INF-γ-stimulated BMDMs, while pretreatment with 0.5 and 1.0 μg/mL CeNP-PEG decreased CD86 expression by 13.1% and 17.1%, respectively (Fig. 4B). Western blotting analysis showed that the protein levels of the proinflammatory marker iNOS and the proinflammatory cytokines TNF-α, IL-6, IL-1β, and IFN-γ were markedly elevated by LPS + IFN-γ stimulation, and this effect was significantly abrogated by the treatment with CeNP-PEG (Fig. 4C, Additional file 1: Fig. S8). Quantitative real-time polymerase chain reaction (qRT-PCR) analysis exhibited that the gene transcription of aforementioned cytokines was markedly increased after stimulation with LPS + INF-γ, and was substantially downregulated by 89.1%, 90.0%, 54.9%, and 97.4% respectively upon pretreatment with 1.0 μg/mL CeNP-PEG (Fig. 4D). Enzyme-linked immunosorbent assay (ELISA) analysis showed similar results: stimulation with LPS + INF-γ induced significant increases in the levels of these cytokines, while 1.0 μg/mL CeNP-PEG strongly suppressed their production by 48.8%, 62.3%, 39.7%, and 47.5%, respectively (Fig. 4E). Collectively, these results demonstrated that CeNP-PEG attenuated the proinflammatory phenotype of macrophage and reduced the production of proinflammatory cytokines in BMDMs.

### In vivo therapeutic effects of CeNP-PEG treatment

**Fig. 5:**
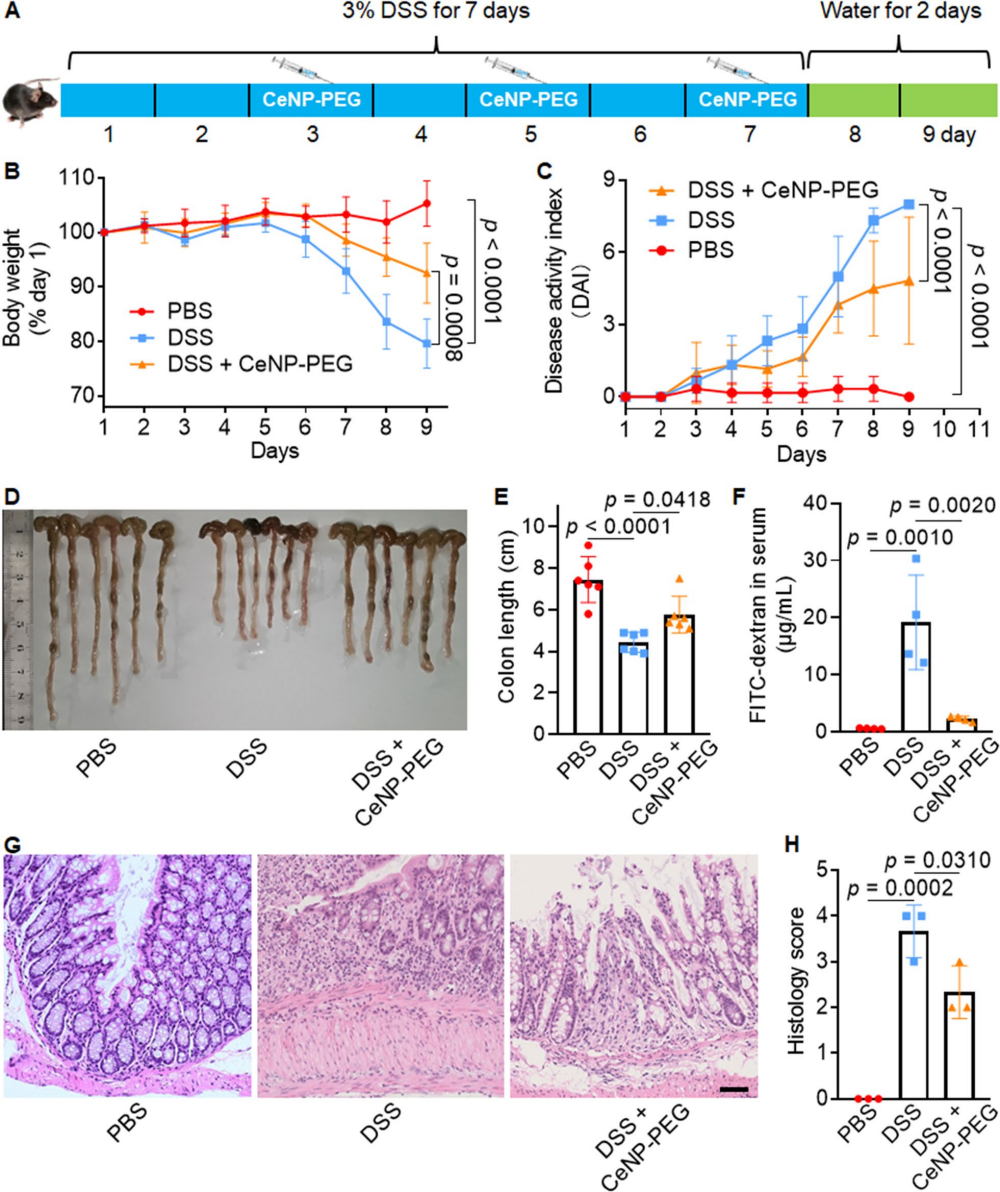
CeNP-PEG exhibited a therapeutic effects on a DSS-induced colitis model. **A** Schematic diagram of colitis mouse model establishment and CeNP-PEG treatment strategies. **B** Normalized mouse body weights as a function of time during the experiment (n = 6). **C** Disease activity index (DAI) score of colitis model mice during the experiment (n = 6). **D** Photographs of the excised colons from the colitis models at day 9 after treatment. **E** Measurements of colon lengths after different treatments (n = 6). **F** Intestinal permeability was examined by measuring the concentration of FITC-dextran in the serum (n = 4). **G** Representative H&E-stained colonic sections at day 9 after treatment. Scale bar = 50 μm. **H** The histology score of colitis in the models after different treatments (n = 3). The data are presented as the mean ± SD. One-way ANOVA was used for statistical analysis

Based on the ex vivo antioxidant and anti-inflammatory effect of CeNP-PEG treatment, the in vivo therapeutic efficacy was investigated using a DSS-induced IBD model of acute colitis in C57BL/6 mice. All mice were divided into one of the following three groups: PBS (not exposed to DSS), DSS exposed, and DSS exposed and CeNP-PEG treated mice (DSS + CeNP-PEG). Figure 5A shows the time course of the overall study procedure. Mice in DSS and DSS + CeNP-PEG groups were given 3% (w/v) DSS in drinking water for 7 consecutive days to induce colitis, followed by plain water for 2 days, while PBS group were given plain water during the experiment. CeNP-PEG was administered to mice in DSS + CeNP-PEG group intravenously on days 3, 5, and 7 at a dose of 1 mg/kg Ce. Compared with the initial weight, body weight in the DSS group decreased by approximately 20%, while body weight in the DSS + CeNP-PEG group was reduced by ~ 8% on day 9 (Fig. 5B). The disease activity index (DAI) score of the DSS + CeNP-PEG group was 60% lower than the DSS group on day 9 (Fig. 5C). Gross observation (Fig. 5D) and the length (Fig. 5E) of the excised colons showed that the average colon length in the PBS and DSS groups were 7.45 ± 1.10 and 4.43 ± 0.48 cm, respectively. The colon length in the DSS + CeNP-PEG group was 5.77 ± 0.88 cm on day 9.

The intestinal permeability was determined by oral administration of FITC-dextran. The FITC-dextran concentration in the blood was 0.56 ± 0.07, 19.16 ± 8.29, and 2.26 ± 0.48 μg/mL in the PBS, DSS and DSS + CeNP-PEG groups, respectively (Fig. 5F). The ROS level in colon was evaluated by dihydroethidium (DHE) staining. The mean fluorescence intensity was 12.27 ± 0.95, 35.31 ± 6.80, and 19.60 ± 1.22 in the PBS, DSS, and DSS + CeNP-PEG groups, respectively (Additional file 1: Fig. S9). Histological analysis showed the inhibitory effect of CeNP-PEG on colonic inflammation, as indicated by the retained the mucosal structure, the restored colonic epithelium, and the reduction in immune cell infiltration compared with those in the DSS group (Fig. 5G). A colon damage scoring system was used to quantify the histological changes. The DSS group had the highest histological score, while CeNP-PEG treatment decreased the histological score by 36.3% (Fig. 5H). These results demonstrated the in vivo efficacy of CeNP-PEG for the amelioration of IBD induced by DSS.

### In vivo regulation of the proinflammatory microenvironment post CeNP-PEG treatment

**Fig. 6:**
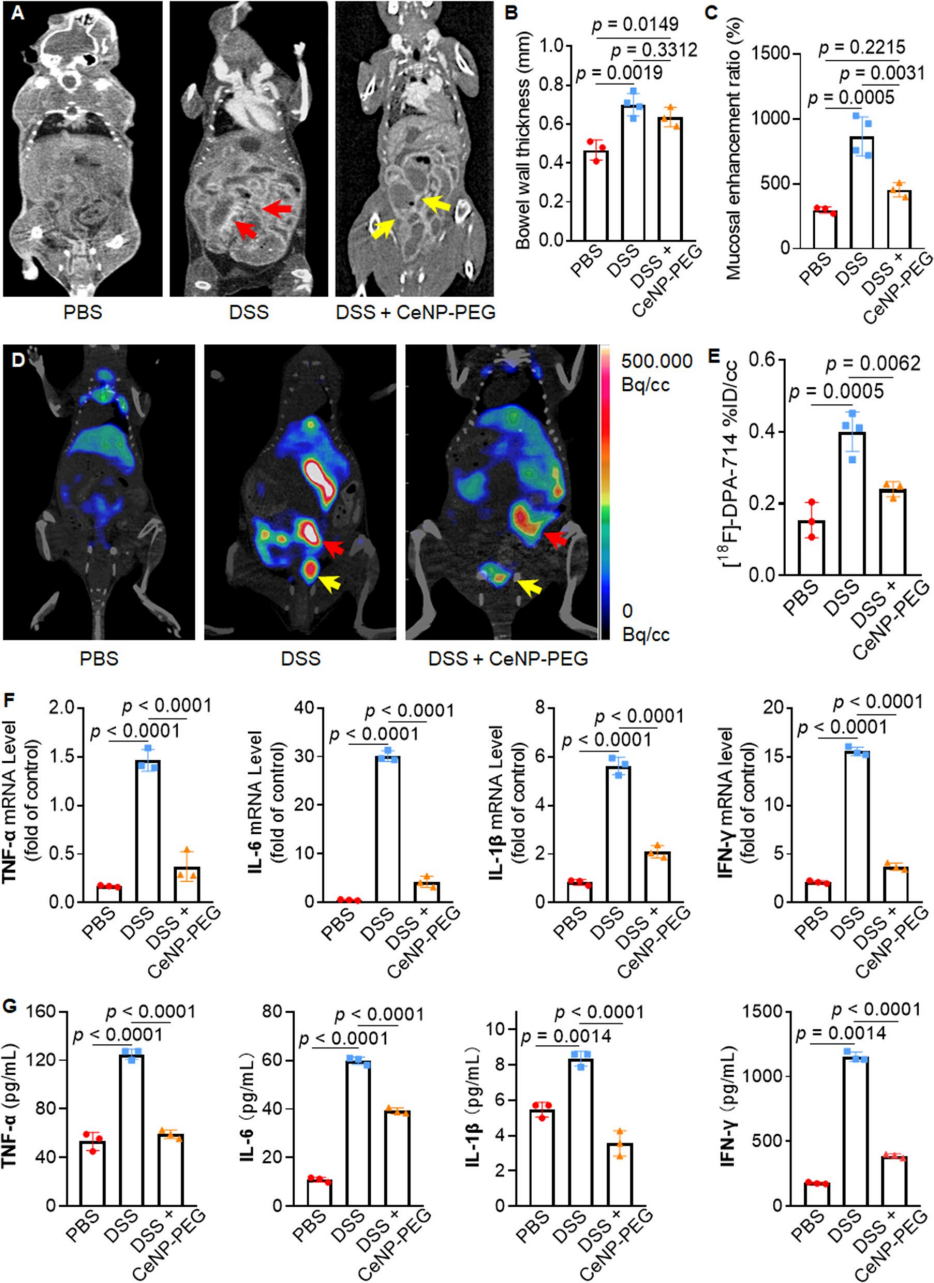
CeNP-PEG rebalanced the proinflammatory microenvironment in the colitis models. **A** Representative CTE images of colitis models after treatment with PBS, 3% DSS and 3% DSS + CeNP-PEG. Segmental bowel wall thickening with mucosal enhancement in DSS-induced colitis models is indicated by red arrows. The alleviation of symtoms after CeNP-PEG treatment is indicated by yellow arrows. **B** The bowel wall thickness was measured by CT (PBS and DSS + CeNP-PEG groups n = 3, DSS group n = 4). **C** Musocal enhancement ratio (the ratio of venous stage enhancement to plain scan) was measured by CT (PBS and DSS + CeNP-PEG groups n = 3, DSS group n = 4). **D** PET/CT images of intestinal inflammatory tissues visualized by the radiotracer [^18^F]-DPA-714 on day 9 after CeNP-PEG treatment. Uptake in the small intestines (red arrow) and distal colon (yellow arrow) in colitis models was attenuated (red and yellow arrows) after CeNP-PEG treatment. **E** Quantification of [^18^F]-DPA-714 radioactivity in the colon (PBS and DSS + CeNP-PEG groups n = 3, DSS group n = 4). **F** The mRNA levels of TNF-α, IL-6, IL-1β, and IFN-γ in colonic tissues on day 9 (n = 3). **G** The serum levels of TNF-α, IL-6, IL-1β, and IFN-γ in colitis model mice on day 9 (n = 3). The data are presented as the mean ± SD. One-way ANOVA was used for statistical analysis

The noninvasive imaging techniques CTE and [^18^F] DPA-714 dynamic PET/CT imaging were used to evaluate intestinal inflammation in IBD. CTE showed segmental thickened bowel walls and luminal dilation in the DSS group, relative to both the PBS and DSS + CeNP-PEG groups (Fig. 6A–C). [^18^F] DPA-714 dynamic PET/CT imaging allows for the quantification of intestinal inflammation as the [^18^F] DPA-714 tracer binds to translocator protein (TSPO). TSPO is overexpressed in activated proinflammatory macrophages and has been used as a hallmark of inflammation and recent studies have shown that [^18^F] DPA-714 is suitable for studying the localized inflammation associated with rodent IBD models [24–27]. The results from the current study clearly demonstrated the difference in intestinal inflammation among the three groups (Fig. 6D and E). When comparing the summed averages over 30–60 min post injection, the [^18^F] DPA-714 concentration observed for the DSS group was significantly greater that the concentrations observed for the PBS and DSS + CeNP-PEG groups (Fig. 6E). Additionally, the ex vivo biodistribution of [^18^F] DPA-714 in the intestines showed a significant difference between treated and untreated mice although its biodistribution in the heart, liver, spleen, lung, and kidney was comparable among the three group (Additional file 1: Fig. S10). The qRT-PCR analysis revealed that the mRNA levels of TNF-α, IL-6, IL-1β, and IFN-γ were markedly enhanced in the DSS group. Their levels were diminished by 74.5%, 86.2%, 62.8%, and 76.1%, however, following CeNP-PEG treatment (Fig. 6F). ELISA confirmed the qRT-PCR results in that CeNP-PEG treatment led to a 52.7%, 34.5%, 57.4%, and 66.4% reduction in the above cytokine concentrations, respectively (Fig. 6G). Moreover, western blotting showed that CeNP-PEG decreased the protein levels of not only the above proinflammatory cytokines but also the proinflammatory phenotypic marker iNOS in mice with DSS-induced colitis (Additional file 1: Fig. S11). Taken together, our data strongly suggest that CeNP-PEG treatment alleviated intestinal inflammation and disease activity by ameliorating the proinflammatory microenvironment.

### CeNP-PEG modulation of proinflammatory macrophages, the Th1/Th17 response and inhibition of the NF-κB and JAK2/STAT3 pathways

**Fig. 7:**
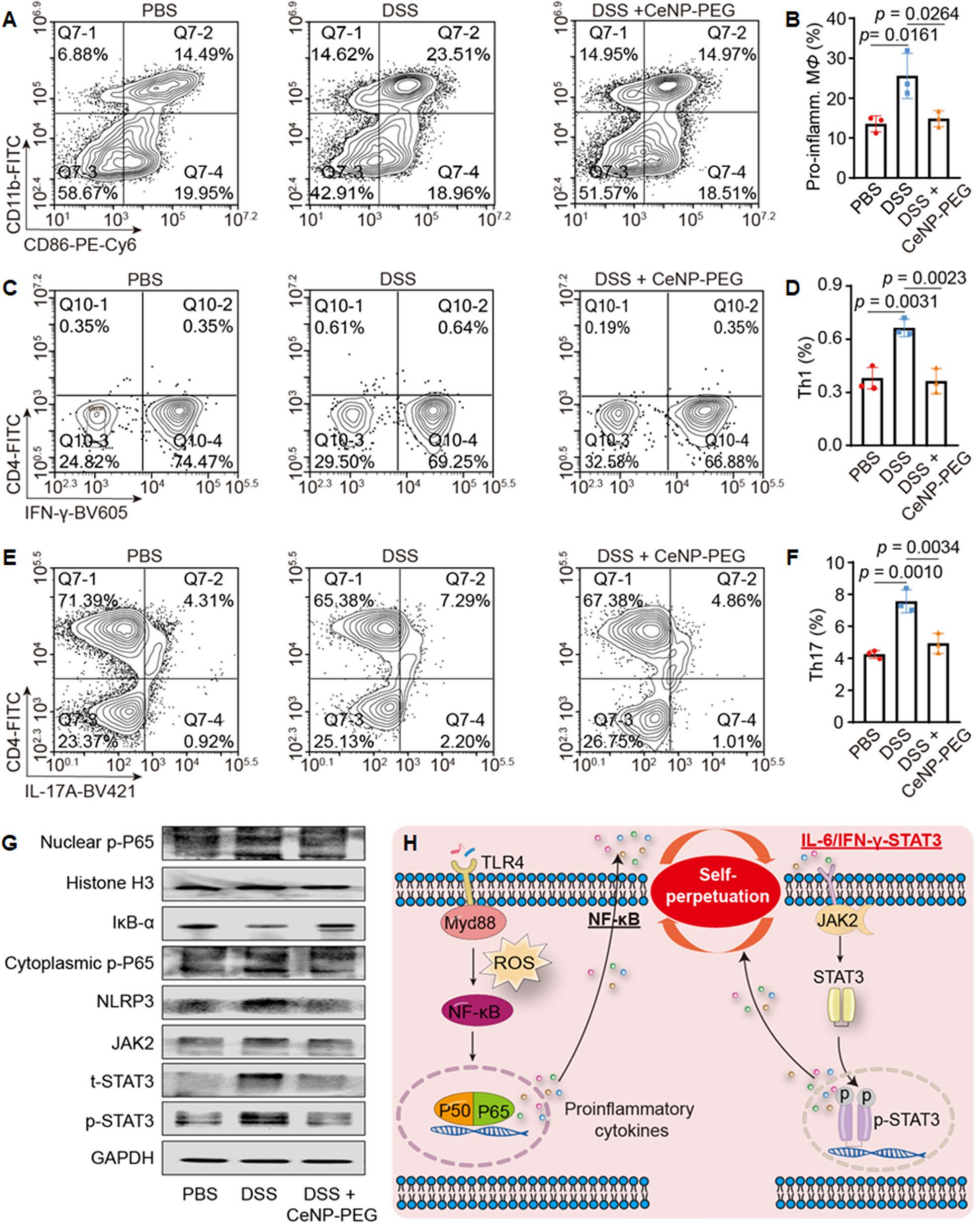
CeNP-PEG exerted a therapeutic effect on colitis model mice by ameliorating the hostile proinflammatory microenvironment. **A** Flow cytometric analysis of proinflammatory macrophages (CD11b^+^ CD86^+^) isolated from colonic tissues on day 9 after different treatments. **B** Quantified percentages of proinflammatory macrophages (n = 3). Proinflamm. M*Φ*: Proinflammatory macrophage. **C** Flow cytometric analysis of Th1 cells (CD4^+^ IFN-γ^+^) isolated from colonic tissues on day 9 after treatment. **D** Quantified percentages of Th1 cells (n = 3). **E** Flow cytometric analysis of Th17 cells (CD4^+^ IL-17A^+^) isolated from colonic tissues on day 9 after treatment. **F** Quantified percentages of Th17 cells (n = 3). **G** Western blotting analysis of key proteins in the NF-κB and JAK2/STAT3 signaling pathways on day 9 after treatmengt. **H** Proposed signaling pathways of self-perpetuation of proinflammatory microenvironment in IBD. The extravasated gut microbiota mediates activation of the NF-κB pathway, which then stimulates the secrete a large amount of proinflammatory cytokines. The elevated IL-6/IFN-γ activates the JAK2/STAT3 pathway, which in turn secretes more proinflammatory cytokines, facilitates the self-perpetuation of proinflammatory microenvironment and promotes IBD progression. The data are presented as the mean ± SD. One-way ANOVA was used for statistical analysis

Flow cytometry showed that the percentages of CD11b^+^ CD86^+^ proinflammatory macrophages sharply increased from 13.56 ± 2.03% in the PBS group to 25.57 ± 5.64% in the DSS groups and were greatly suppressed to 14.85 ± 2.00% in response to CeNP-PEG treatment (Fig. 7A and B, Additional file 1: Fig. S12). Immunohistochemical staining revealed infiltration of proinflammatory macrophages that coexpressed with CD11b^+^ and CD86^+^ and epithelial damage in the colon of the DSS group, relative to the PBS group. CeNP-PEG treatment profoundly restricted infiltration and subsequent epithelial damage (Additional file 1: Fig. S13). Since Th1/Th17 subsets are involved in exacerbating the inflammatory response in a DSS-induced colitis model [3, 28], we assessed whether CeNP-PEG could affect the differentiation of helper CD4^+^ T cells. Flow cytometric analysis showed 0.38 ± 0.06%, 0.66 ± 0.05%, and 0.36 ± 0.07% CD4^+^ IFN-γ^+^ Th1 cells and 4.25 ± 0.25%, 7.56 ± 0.72%, and 4.9 ± 0.64% CD4^+^ IL17A^+^ Th17 cells in the PBS, DSS, and DSS + CeNP-PEG groups, respectively. This finding suggests that CeNP-PEG may restrain the proinflammatory differentiation of CD4^+^ T cells (Fig. 7C–F, Additional file 1: Figs. S14 and S15).

There is growing evidence that NF-κB and JAK2/STAT3 signaling pathways play an important role in the pathogenesis of IBD [29, 30]. Thus, we assessed changes in the signaling pathways of NF-κB and JAK2/STAT3 using western blotting analysis. The results showed that while protein levels of IκB-α decreased in the DSS group, a dramatic upregulation of the phosphorylation of cytoplasmic P65 (cytoplasmic p-P65) and nuclear translocation (nuclear p-P65) was observed. Increased the levels of JAK2, total STAT3 (t-STAT3) and phosphorylated STAT3 (p-STAT3) were observed in the DSS group, thereby suggesting activation of the NF-κB and JAK2/STAT3 pathways in IBD progression (Fig. 7G, Additional file 1: Fig. S16). Additionally, it is known that the NLR family pyrin domain-containing 3 (NLRP3) protein is critical for controlling inflammasome activation [31, 32]. NLRP3 was noticeably augmented in the DSS group (Fig. 7G, Additional file 1: Fig. S16). However, in the DSS + CeNP-PEG group the level of IκB-α was much higher than that in the DSS group, and the abovementioned key signaling proteins of the NF-κB and JAK2/STAT3 pathways were recovered to levels comparable to those in the PBS group. The results clearly indicate that CeNP-PEG treatment markedly suppressed activation of the NF-κB and JAK2/STAT3 pathways (Fig. 7G, Additional file 1: Fig. S16). Based on the previous reports and our experimental data, we proposed that the extravasated gut microbiota that mediates the inflammatory response produces excess ROS and activates the NF-κB pathway. The NF-κB pathway secretes a robust amount of proinflammatory cytokines such as TNF-α, IL-6, and IFN-γ [33, 34]. IL-6 and IFN-γ then induces the activation of JAK2/STAT3, resulting in widespread inflammatory response [29]. The concomitant inflammatory cascades of NF-κB and JAK2/STAT3 mediated pathway further argument NF-κB activity and establish an self-perpetuation inflammatory circuit to exacerbate IBD (Fig. 7H). Additionally, the biosafety of CeNP-PEG was evaluated by organ index measurement and H&E staining of major organs. We did not observe abnormal differences in the organ indexes or histopathological changes in the major organs in each group (Additional file 1: Fig. S17).

## Discussion

Conventional IBD treatment suffers from limited efficacy, high unresponsive rates, systemic side effects, and there is a need to develop novel treatment options [19, 35]. Intestinal macrophages are critical in establishing and maintaining gut homeostasis and are key mediators of the immune response during gut inflammation due to their considerable functional plasticity and multifaceted roles in response to environment signals [6, 7]. Macrophages, which are the main cellular components in the IBD proinflammatory microenvironment, adopt a proinflammatory phenotype and secrete various proinflammatory cytokines, chemokines, and ROS, which contribute to the progression of IBD [11, 12]. Retrospective analysis of clinical data demonstrated that IBD patients with higher disease severity were prone to have higher infiltration of intestinal proinflammatory macrophages and higher serum levels of proinflammatory cytokines, strongly implying the regulatory effect of the intestinal proinflammatory macrophages on the disease severity in IBD patients. It has been well documented that ROS are essential for the induction and maintenance of proinflammatory phenotypes and promote proinflammatory gene expression in macrophages [23]. In addition to the modulatory effect on macrophages, the continued increase of ROS triggers the interplays between immune and non-immune cells which forms a self-perpetuating microenvironment that sustains the inflammation and accelerate the injury of surrounding tissues [14]. Thus, the self-perpetuating proinflammatory microenvironment can somehow explain the chronic and relapse characteristics of IBD. Therefore, ROS scavengers that eliminate excess ROS will disrupt the vicious cycle to ameliorate and rebalance the proinflammatory microenvironment.

Recently, nanozymes with potent antioxidant effects have attracted considerable attention and have been widely used in the treatment of ROS-associated diseases such as IBD, liver injury, and acute kidney injury [15, 17, 36]. Compared with traditional small molecule antioxidants or natural enzymes, which suffer from the intrinsic disadvantages of stoichiometric action, high substrate specificity, and nonregenerative activity, the drug-free nanozyme CeNP-PEG shows superior regenerative antioxidant capability and can simultaneously eradicate multiple types of ROS [16, 18, 37]. In this study, we prepared ultrasmall drug-free ceria nanozyme with broad-spectrum ROS scavenging capacity, outstanding anti-inflammatory activity, and excellent biocompatibility. The naked CeNP with diameter less than 5 nm are more favorable to ensure the high pseudo-enzyme activity, and the average hydrodynamic diameter about 10 nm after PEGylation indicated the formation of monodisperse CeNP-PEG with excellent colloidal stability [16, 38]. The PEGylation of CeNP provides “stealth” characteristics, which improves the colloidal stabilization and prolongs the circulation time in the blood stream by reducing nonspecific binding and uptake by the mononuclear phagocyte system [39]. The smaller size of CeNP-PEG enables their facile cellular uptake by endocytosis and/or transmembrane fusion, whereas larger size may affect their cellular uptake pathway and biodistribution in vivo [40–43]. Our results demonstrated that CeNP-PEG possessed robust •OH-scavenging effects and SOD- and CAT-mimetic activities. Holding the multi-enzymatic activities against ROS, CeNP-PEG are of tremendous potential to protect cells from several forms of cellular damage. After being taken up by BMDMs, CeNP-PEG efficiently scavenge intracellular ROS, dramatically reduce the gene transcription and protein levels of proinflammatory cytokines, and inhibit the proinflammatory phenotypic transition of BMDMs. Moreover, our results showed that CeNP-PEG treatment diminished weight loss, decreased DAI scores, restrained colon length shortening, improved intestinal permeability, reduced colonic ROS levels, and restored disruption in the colonic epithelium and mucosal structure in DSS-induced IBD model mice.

CTE is a useful diagnostic technique for assessing inflammation and monitoring disease activity of IBD that can provide real biological phenomena that correlate with inflammation [44, 45]. Segmental thickened bowel wall and strong mucosal enhancement are the two most sensitive signs of active IBD. Thickened bowel wall with layering enhancement is predictive of acute disease, while homogeneous enhancement suggests quiescence. Our results demonstrated the decreased bowel wall thickness accompanied by the significant attenuation of mucosal enhancement in colitis mice receiving CeNP-PEG treatment, suggesting the mitigation of colitis severity in response to CeNP-PEG treatment. Furthermore, PET/CT imaging provides a novel opportunity to monitor the dynamic transformation of macrophages and track their function. TSPO is overexpressed in activated macrophages and can serve as a biomarker of inflammation [46]. PET/CT imaging allows for the quantification of inflammation with radioligands [^18^F]-DPA-714 that specifically bind to TSPO [26]. We demonstrated significantly higher uptake and biodistribution of [^18^F]-DPA-714 in the intestine in the DSS group than in the CeNP-PEG group, suggesting that more proinflammatory macrophages accumulated in the intestine in the DSS group than in the CeNP-PEG group. Moreover, the mRNA expression level in colonic tissues and serum concentration of proinflammatory cytokines were markedly downregulated post CeNP-PEG treatment.

Mounting researches have been proved that innate and adaptive immunity such as macrophages and T helper cells (Th1 and Th17 cells) are both involve in IBD pathogenesis [3, 28]. Flow cytometry analysis of colonic tissues revealed that treatment with CeNP-PEG profoundly decreased the number of proinflammatory microphages and Th1/Th17 cells in DSS-induced colitis, which may also contribute the dampened proinflammatory microenvironment. The co-activation of NF-κB and JAK2/STAT3 induce a massive and sustained production of proinflammatory cytokines, such as IL-6 and IFN-γ, which are critical for the development of inflammatory diseases including rheumatoid arthritis (RA) and multiple sclerosis (MS) [22, 47]. Notably, the co-activation of NF-κB and JAK2/STAT3 observed in this study may drive the perpetuation of the proinflammatory microenvironment and CeNP-PEG treatment markedly suppressed consistent co-activation of the NF-κB and JAK2/STAT3 pathways, which ameliorate the inflammatory microenvironment in IBD.

In line with previous reports showing that ceria nanoparticles were nontoxic in vivo at the therapeutic doses, we did not observe any toxicity of CeNP-PEG in current study [48]. The biodistribution of CeNP-PEG in our previous study indicated that they mainly accumulate in the liver and spleen after intravenous injection, the quantitative analysis of ICP-AES showed that the uptake of CeNP-PEG reached a peak at 2 h and subsequently decreased [49], indicating that the possible clearance pathway of CeNP-PEG was reticuloendothelial system (RES) and then excretion through feces [50]. Some studies have been reported that ceria nanoparticles in the liver were found to decrease their size over time, suggesting the possible biodegradability process of ceria nanoparticles [51, 52].

## Conclusions

In summary, we developed the ultrasmall drug-free nanozyme CeNP-PEG with multiple ROS scavenging activities for the efficient treatment of IBD. CeNP-PEG possess robust •OH, O_2_^•−^ and H_2_O_2_ scavenging capacities, clearly reduce the levels of multiple proinflammatory cytokines and the proinflammatory features of macrophages. Furthermore, CeNP-PEG significantly attenuated the intestinal inflammatory microenvironment in a DSS-induced IBD model by decreasing the proinflammatory responses of macrophages and Th1/Th17 cells. The molecular mechanism by which CeNP-PEG protect against IBD may be attributed to suppression the co-activation of the NF-κB and JAK2/STAT3 pathways to ameliorate the proinflammatory microenvironment. Our study not only helps to explain the therapeutic mechanism of CeNP-PEG for IBD treatment but also provides a new strategy to address the unsatisfactory clinical requirement of treating inflammation-related diseases by ameliorating the proinflammatory microenvironment.

## Supplementary Information

**Additional file 1.** Supplementary Material.

## Data Availability

All data generated or analyzed during this study is available from the corresponding author on reasonable request.

## Abbreviations

IBD: Inflammatory bowel disease
ROS: Reactive oxygen species
DSS: Dextran sulfate sodium
CTE: Computed tomographic enterography
PET/CT: Positron emission tomography/computed tomographic
STAT3: Signal transducer and activator of transcription-3
JAK2: Janus kinase 2
NF-κB: Nuclear factor kappa-B
UC: Ulcerative colitis
CD: Crohn’s disease
CeNP: Ceria nanoparticles
CeNP-PEG: Polyethylene glycol-capped ultrafine ceria nanoparticles
IL-1β: Interleukin-1β
IL-6: Interleukin-6
TNF-α: Tumor necrosis factor-α
IFN-γ: Interferon-γ
H_2_O_2_: Hydrogen peroxide
•OH: Hydroxyl radicals
O_2_^•^^−^: Superoxide anions
SOD: Superoxide dismutase
CAT: Catalase
Th1: T helper 1
Th17: T helper 17
CRP: C-reactive protein
UCEIS: Ulcerative colitis endoscopic index of severity
DAPI: 4,6-Diamidino-2-phenylindole
TEM: Transmission electron microscopy
EDS: Energy-dispersive spectroscopy
SAED: Selected-area electron diffraction
XRD: X-ray diffraction
XPS: X-ray photoelectron spectroscopy
PDI: Polymer dispersity index
BMDMs: Bone marrow-derived macrophages
ESR: Electron spin resonance
DMPO: 5,5-Dimethyl-1-pyrroline N-oxide
CTPO: 3-Carbamoyl-2,5-dihydro-2,2,5,5-tetramethyl-1H-pyrrol-1-yloxyl
DCF: 2’, 7’-Dichlorodihydrofluorescein
qRT-PCR: Quantitative real-time polymerase chain reaction
ELISA: Enzyme-linked immunosorbent assay
TSPO: Translocator protein
DAI: Disease activity index
ANOVA: Analysis of variance
ICP-AES: Inductively coupled plasma-atomic emission spectrometry
RES: Reticuloendothelial system

## References

[1] Kudelka MR, Stowell SR, Cummings RD, Neish AS (2020). Intestinal epithelial glycosylation in homeostasis and gut microbiota interactions in IBD. Nat Rev Gastroenterol Hepatol.

[2] Khor B, Gardet A, Xavier RJ (2011). Genetics and pathogenesis of inflammatory bowel disease. Nature.

[3] Lee B-C, Lee JY, Kim J, Yoo JM, Kang I, Kim J-J, Shin N, Kim DJ, Choi SW, Kim D, Hong BH, Kang K-S (2020). Graphene quantum dots as anti-inflammatory therapy for colitis. Sci Adv.

[4] Nielsen OH (2014). New strategies for treatment of inflammatory bowel disease. Front Med.

[5] Marafini I, Sedda S, Dinallo V, Monteleone G (2019). Inflammatory cytokines: from discoveries to therapies in IBD. Expert Opin Biol Th.

[6] de Souza HSP, Fiocchi C (2016). Immunopathogenesis of IBD: current state of the art. Nat Rev Gastroenterol Hepatol.

[7] Hamilton JA (2008). Colony-stimulating factors in inflammation and autoimmunity. Nat Rev Immunol.

[8] Zhu H, Li YR (2012). Oxidative stress and redox signaling mechanisms of inflammatory bowel disease: updated experimental and clinical evidence. Exp Biol Med.

[9] Rezaie A, Parker RD, Abdollahi M (2007). Oxidative stress and pathogenesis of inflammatory bowel disease: an epiphenomenon or the cause?. Dig Dis Sci.

[10] Pereira C, Grácio D, Teixeira JP, Magro F (2015). Oxidative stress and DNA damage: implications in inflammatory bowel disease. Inflamm Bowel Dis.

[11] Tang Y, Shi Y, Gao Y, Xu X, Han T, Li J, Liu C (2019). Oxytocin system alleviates intestinal inflammation by regulating macrophages polarization in experimental colitis. Clin Sci.

[12] Zhang G, Ma L, Bai L, Li M, Guo T, Tian B, He Z, Fu Q (2021). Inflammatory microenvironment-targeted nanotherapies. J Control Release.

[13] Friedrich M, Pohin M, Powrie F (2019). Cytokine networks in the pathophysiology of inflammatory bowel disease. Immunity.

[14] Zhang B, Bailey WM, McVicar AL, Gensel JC (2016). Age increases reactive oxygen species production in macrophages and potentiates oxidative damage after spinal cord injury. Neurobiol Aging.

[15] Li F, Qiu Y, Xia F, Sun H, Ling D (2020). Dual detoxification and inflammatory regulation by ceria nanozymes for drug-induced liver injury therapy. Nano Today.

[16] Zeng F, Wu Y, Li X, Ge X, Guo Q, Lou X, Cao Z, Hu B, Long NJ, Mao Y, Li C (2018). Custom-made ceria nanoparticles show a neuroprotective effect by modulating phenotypic polarization of the microglia. Angew Chem Int Ed.

[17] Weng Q, Sun H, Fang C, Xia F, Liao H, Lee J, Wang J, Xie A, Ren J, Guo X, Li F, Yang B, Ling D (2021). Catalytic activity tunable ceria nanoparticles prevent chemotherapy-induced acute kidney injury without interference with chemotherapeutics. Nat Commun.

[18] Ni D, Wei H, Chen W, Bao Q, Rosenkrans ZT, Barnhart TE, Ferreira CA, Wang Y, Yao H, Sun T, Jiang D, Li S, Cao T, Liu Z, Engle JW, Hu P, Lan X, Cai W (2019). Ceria nanoparticles meet hepatic ischemia-reperfusion injury: the perfect imperfection. Adv Mater.

[19] Zhao S, Li Y, Liu Q, Li S, Cheng Y, Cheng C, Sun Z, Du Y, Butch CJ, Wei H (2020). An orally administered CeO2@Montmorillonite nanozyme targets inflammation for inflammatory bowel disease therapy. Adv Funct Mater.

[20] Soh M, Kang D-W, Jeong H-G, Kim D, Kim DY, Yang W, Song C, Baik S, Choi I-Y, Ki S-K, Kwon HJ, Kim T, Kim CK, Lee S-H, Hyeon T (2017). Ceria-Zirconia nanoparticles as an enhanced multi-antioxidant for sepsis treatment. Angew Chem Int Ed.

[21] Thangudu S, Su C-H (2021). Peroxidase mimetic nanozymes in cancer phototherapy: progress and perspectives. Biomolecules.

[22] Ota M, Tanaka Y, Nakagawa I, Jiang J-J, Arima Y, Kamimura D, Onodera T, Iwasaki N, Murakami M (2020). Role of chondrocytes in the development of rheumatoid arthritis via transmembrane protein 147–mediated NF-κB activation. Arthr Rheumatol.

[23] Zhao Y, Yang Y, Zhang J, Wang R, Cheng B, Kalambhe D, Wang Y, Gu Z, Chen D, Wang B, Huang Y (2020). Lactoferrin-mediated macrophage targeting delivery and patchouli alcohol-based therapeutic strategy for inflammatory bowel diseases. Acta Pharm Sin B.

[24] Takkinen JS, López-Picón FR, Al Majidi R, Eskola O, Krzyczmonik A, Keller T, Löyttyniemi E, Solin O, Rinne JO, Haaparanta-Solin M (2016). Brain energy metabolism and neuroinflammation in ageing APP/PS1–21 mice using longitudinal 18F-FDG and 18F-DPA-714 PET imaging. J Cereb Blood Flow Metab.

[25] Abourbeh G, Thézé B, Maroy R, Dubois A, Brulon V, Fontyn Y, Dollé F, Tavitian B, Boisgard R (2012). Imaging microglial/macrophage activation in spinal cords of experimental autoimmune encephalomyelitis rats by positron emission tomography using the mitochondrial 18 kDa translocator protein radioligand [18F]DPA-714. J Neurosci.

[26] Guo Q (2018). A TSPO PET imaging study to evaluate inflammation in Crohn's disease. J Nucl Med.

[27] Bernards N, Pottier G, Thézé B, Dollé F, Boisgard R (2015). In vivo evaluation of inflammatory bowel disease with the aid of μPET and the translocator protein 18 kDa radioligand [18F]DPA-714. Mol Imag Biol.

[28] Ueno A, Jeffery L, Kobayashi T, Hibi T, Ghosh S, Jijon H (2018). Th17 plasticity and its relevance to inflammatory bowel disease. J Autoimmun.

[29] Tao J-H, Duan J-A, Zhang W, Jiang S, Guo J-M, Wei D-D (2018). Polysaccharides from chrysanthemum morifolium ramat ameliorate colitis rats via regulation of the metabolic profiling and NF-κ B/TLR4 and IL-6/JAK2/STAT3 signaling pathways. Front Pharmacol..

[30] Wang J, Pan Y, Cao Y, Zhou W, Lu J (2019). Salidroside regulates the expressions of IL-6 and defensins in LPS-activated intestinal epithelial cells through NF-κB/MAPK and STAT3 pathways. Iran J Basic Med Sci.

[31] Wang S, Lin Y, Yuan X, Li F, Guo L, Wu B (2018). REV-ERBα integrates colon clock with experimental colitis through regulation of NF-κB/NLRP3 axis. Nat Commun.

[32] Johnson DE, O'Keefe RA, Grandis JR (2018). Targeting the IL-6/JAK/STAT3 signalling axis in cancer. Nat Rev Clin Oncol.

[33] Chen L, You Q, Hu L, Gao J, Meng Q, Liu W, Wu X, Xu Q (2018). The antioxidant procyanidin reduces reactive oxygen species signaling in macrophages and ameliorates experimental colitis in mice. Front Immunol.

[34] Silva FAR, Rodrigues BL, Ayrizono MLS, Leal RF (2016). The immunological basis of inflammatory bowel disease. Gastroent Res Pract..

[35] Papamichael K, Gils A, Rutgeerts P, Levesque BG, Vermeire S, Sandborn WJ, VandeCasteele N (2015). Role for therapeutic drug monitoring during induction therapy with TNF antagonists in IBD: evolution in the definition and management of primary nonresponse. Inflamm Bowel Dis.

[36] Liu Y, Cheng Y, Zhang H, Zhou M, Yu Y, Lin S, Jiang B, Zhao X, Miao L, Wei C-W, Liu Q, Lin Y-W, Du Y, Butch CJ, Wei H (2020). Integrated cascade nanozyme catalyzes in vivo ROS scavenging for anti-inflammatory therapy. Sci Adv.

[37] Wang Q, Wei H, Zhang Z, Wang E, Dong S (2018). Nanozyme: an emerging alternative to natural enzyme for biosensing and immunoassay, TrAC. Trends Anal Chem.

[38] Li Y, He X, Yin J-J, Ma Y, Zhang P, Li J, Ding Y, Zhang J, Zhao Y, Chai Z, Zhang Z (2015). Acquired superoxide-scavenging ability of ceria nanoparticles. Angew Chem Int Ed.

[39] Karakoti AS, Das S, Thevuthasan S, Seal S (2011). PEGylated inorganic nanoparticles. Angew Chem Int Ed.

[40] Verma A, Uzun O, Hu Y, Hu Y, Han H-S, Watson N, Chen S, Irvine DJ, Stellacci F (2008). Surface-structure-regulated cell-membrane penetration by monolayer-protected nanoparticles. Nat Mater.

[41] Torrano AA, Herrmann R, Strobel C, Rennhak M, Engelke H, Reller A, Hilger I, Wixforth A, Bräuchle C (2016). Cell membrane penetration and mitochondrial targeting by platinum-decorated ceria nanoparticles. Nanoscale.

[42] Kwon HJ, Kim D, Seo K, Kim YG, Han SI, Kang T, Soh M, Hyeon T (2018). Ceria nanoparticle systems for selective scavenging of mitochondrial, intracellular, and extracellular reactive oxygen species in Parkinson's Disease. Angew Chem Int Ed.

[43] Singh S, Ly A, Das S, Sakthivel TS, Barkam S, Seal S (2018). Cerium oxide nanoparticles at the nano-bio interface: size-dependent cellular uptake. Artif Cells Nanomed Biotechnol.

[44] Hashimoto S, Shimizu K, Shibata H, Kanayama S, Tanabe R, Onoda H, Matsunaga N, Sakaida I (2013). Utility of computed tomographic enteroclysis/enterography for the assessment of mucosal healing in Crohn's disease. Gastroent Res Pract..

[45] Higgins PDR, Caoili E, Zimmermann M, Bhuket TP, Sonda PL, Manoogian B, Platt JF, Zimmermann EM (2007). Computed tomographic enterography adds information to clinical management in small bowel Crohn's disease. Inflamm Bowel Dis.

[46] Thiel A, Heiss W-D (2011). Imaging of microglia activation in stroke. Stroke.

[47] Ogura H, Murakami M, Okuyama Y, Tsuruoka M, Kitabayashi C, Kanamoto M, Nishihara M, Iwakura Y, Hirano T (2008). Interleukin-17 promotes autoimmunity by triggering a positive-feedback loop via interleukin-6 induction. Immunity.

[48] Casals E, Zeng M, Parra-Robert M, Fernández-Varo G, Morales-Ruiz M, Jiménez W, Puntes V, Casals G (2020). Cerium oxide nanoparticles: advances in biodistribution, toxicity, and preclinical exploration. Small.

[49] Wang M, Zeng F, Ning F, Wang Y, Zhou S, He J, Li C, Wang C, Sun X, Zhang D, Xiao J, Hu P, Reilly S, Xin H, Xu X, Zhang X (2022). Ceria nanoparticles ameliorate renal fibrosis by modulating the balance between oxidative phosphorylation and aerobic glycolysis. J Nanobiotechnol.

[50] Park K, Park J, Lee H, Choi J, Yu W-J, Lee J (2018). Toxicity and tissue distribution of cerium oxide nanoparticles in rats by two different routes: single intravenous injection and single oral administration. Arch Pharm Res.

[51] Graham UM, Tseng MT, Jasinski JB, Yokel RA, Unrine JM, Davis BH, Dozier AK, Hardas SS, Sultana R, Grulke EA, Butterfield DA (2014). In vivo processing of ceria nanoparticles inside liver: impact on free-radical scavenging activity and oxidative stress. ChemPlusChem.

[52] Modrzynska J, Berthing T, Ravn-Haren G, Kling K, Mortensen A, Rasmussen RR, Larsen EH, Saber AT, Vogel U, Loeschner K (2018). In vivo-induced size transformation of cerium oxide nanoparticles in both lung and liver does not affect long-term hepatic accumulation following pulmonary exposure. PLoS ONE.

