# Supplementary material for: A drug-free nanozyme for mitigating oxidative stress and inflammatory bowel disease
Source: J Nanobiotechnology. 2022 Mar 4;20:107. doi: 10.1186/s12951-022-01319-7 (PMC8896226; doi:10.1186/s12951-022-01319-7)
Supplement: Supplementary file 1 — Additional file 1. Supplementary Material. [file 12951_2022_1319_MOESM1_ESM.docx]

**Supplementary Material**

**A drug-free** **nanozyme for mitigating oxidative stress and inflammatory bowel disease**

Feng Zeng^#a^, Yahong Shi^#b^, Chunni Wu^a^, Jianming Liang^a^, Qixin Zhong^o^, Karen Briley^p^, Bin Xu^n^, Yongzhuo Huang^e ,f, g^, Manmei Long ^k^, Cong Wang ^c, h^, Jian Chen ^c^, Yonghua Tang ^m^, Xinying Li ^b^, Mengda Jiang ^b^, Luting Wang ^e^, Qin Xu^a^, Liu Yang ^l^, Peng Chen^a^, Shengzhong Duan^i,j^, Jingyuan Xie* ^d^, Cong Li* ^c^, Yingwei Wu* ^b^

^a^Artemisinin Research Center, Institute of Science and Technology, The First Affiliated Hospital, The first Clinical Medical School, Lingnan Medical Research Center, Guangzhou University of Chinese Medicine, Guangzhou 510450, China

^b^Department of Radiology, Shanghai Ninth People’s Hospital, Shanghai Jiao Tong University School of Medicine, Shanghai 200011, China

E-mail: [wuyw0103@hotmail.com](mailto:wuyw0103@hotmail.com)

^c^Key Laboratory of Smart Drug Deliver, Ministry of Education, School of Pharmacy, Fudan University, Shanghai 201213, China

E-mail: [congli@fudan.edu.cn](mailto:congli@fudan.edu.cn)

^d^Department of Nephrology, Institute of Nephrology, Shanghai Ruijin Hospital, Shanghai Jiao Tong University School of Medicine, Shanghai 200020, China.

E-mail: [xielicheng0127@163.com](mailto:xielicheng0127@163.com)

^e^Shanghai Institute of Materia Medica, Chinese Academy of Sciences, Shanghai 201203, China

^f^Zhongshan Institute for Drug Discovery and Development, Chinese Academy of Sciences, Zhongshan 528437, China

^g^Taizhou University, School of Advanced Study, Institute of Natural Medicine and Health Product, Taizhou 318000, China

^h^China Academy for Engineering and Technology, Fudan University, Shanghai 200433, China

^i^Laboratory of Oral Microbiota and Systemic Diseases, Shanghai Ninth People's Hospital, College of Stomatology, Shanghai Jiao Tong University School of Medicine, Shanghai 200125, China.

^j^National Clinical Research Center for Oral Diseases, Shanghai Key Laboratory of Stomatology and Shanghai Research Institute of Stomatology, Shanghai 200011, China

^k^Department of Pathology, Shanghai Ninth People’s Hospital, Shanghai Jiao Tong University School of Medicine, Shanghai 200011, China

^l^Department of Molecular Diagnostics, The Core Laboratory in Medical Center of Clinical Research, Department of Endocrinology, Shanghai Ninth People’s Hospital, State Key Laboratory of Medical Genomics, Shanghai Jiao Tong University School of Medicine, Shanghai 200011, China

^m^Radiology Department, Ruijin Hospital, Shanghai Jiao Tong University School of Medicine, Shanghai 200020, China

^n^Department of Gastroenterology, Ruijin Hospital, Shanghai Jiao Tong University School of Medicine, Shanghai 200020, China

^o^Department of Cardiovascular, Shenzhen Hospital of Guangzhou University of Chinese Medicine, Shenzhen 518034, China

^p^Invicro, A Konica Minolta Company, Boston, MA 02210, United States

[^#^] These authors contributed equally to this work.

**Materials and reagents**

Cerium (III) acetate hydrate, oleylamine were purchased form Adamas-beta, Titan Scientific Co., Ltd. (Shanghai, China). Xylenes, hydrogen peroxide (H_2_O_2_, 30%), chloroform, methanol, nitric acid (HNO_3_, 65-68%), Tween 20, Diethylenetriaminepentaacetic acid (DTPA), FeSO_4_·7H_2_O, Na_2_HPO_4_·12H_2_O, and NaH_2_PO_4_·2H_2_O were obtained from Sinopharm Chemical Reagent Co., Ltd. (Shanghai, China). 5 (6)-Carboxy-tetramethylrhodamine N-succinimidyl ester (5 (6)-TAMRA SE) was purchased from Dibo Chemical Technology Co., Ltd. (Shanghai China). Hypoxantine, Xanthine oxidase, 5,5-dimethyl-1-pyrroline N-oxide (DMPO) was purchased from Dojindo Laboratories (Kumamoto, Japan). 3-Carbamoyl-2,5-dihydro-2,2,5,5-tetramethyl-1*H*-pyrrol-1-yloxyl (CTPO) was purchased from J&K Scientific Ltd (Beijing, China). Recombinant murine macrophage colony stimulating factor (M-CSF), Recombinant murine interferon-gamma (IFN-γ) were acquired from Novoprotein Scientific Inc. (Suzhou, China). Lipopolysaccharide (LPS), Dulbecco’s Modified Eagle Medium (DMEM) and Cell counting kit-8 (CCK-8) was obtained from Meilunbio Co., Ltd. (Dalian, China). ROS assay kit, nuclear and cytoplasmic protein extraction kit, RIPA lysis buffer, and bicinchoninic acid (BCA) protein assay kit were purchased from Beyotime Institute of Biotechnology (Haimen, China). The mPEG^2k^-DPSE, NH_2_-PEG^2k^-DPSE were acquired from huateng pharmaceutical co., Ltd. (Hunan, China).

**IBD** **patient examination data and colon specimens**

The coronal computed tomographic enterography (CTE) and endoscopic data of healthy control (n = 10 for CTE and n = 5 for endoscopic), mild UC patients (n = 10 for both CTE and endoscopic), and severe UC patients (n = 10 for CTE and n = 15 for endoscopic) were obtained from patients presented at Shanghai Ninth People’s Hospital to identify the radiological and endoscopic disease severity, respectively. In addition, the serum test results of patients with mild and severe UC (n = 10 mild UC and n = 18 severe UC) were acquired in the medical record from Shanghai Ninth People’s Hospital. Human colon tissues were collected from patients with IBD who underwent surgical resection at Shanghai Ninth People’s Hospital between 2018 and 2021. The guidelines approved by the Shanghai Ninth People’s Hospital Institutional Review Board were followed and written informed consents were obtained from all patients. Colonic tissues were excised from four patients (four males, age range: 29-56 years). Histologic examination was carried out by two senior pathologists. Both inflammation lesions and adjacent normal tissues were used for immunofluorescence staining with pro-inflammatory macrophage phenotype biomarkers CD11b and CD86.

**Synthesis of CeNP**

The ceria nanoparticles were synthesized according to previously reported procedures with slight modifications.^[^[^1^](#_ENREF_1)^]^ Firstly, cerium (III) acetate hydrate (0.43 g, 1.0 mmol) and oleylamine (15 mL) were dispersed in xylenes (15 mL) and sonicated until the solution became clear and transparent. After slowly heating to 110 °C under N_2_ protection, ddH_2_O (1 mL) was injected into the solution swiftly under vigorous stirring, and the mixture was stirred and aged at 110 °C for 3 h in N_2_ atmosphere. The CeNP was precipitated by adding methanol after cooling down to room temperature and then harvested by centrifugation at 10000 rpm for 10 min. The purified CeNP was dissolved in chloroform for further reaction.

**Synthesis of CeNP-PEG**

To synthesis biocompatible ceria nanoparticles, CeNP were coated with DSPE-PEG co-polymer via combining film hydration method and probe sonication assisted method.^[^[^2^](#_ENREF_2)^]^ Briefly, mPEG^2k^-DPSE (50 mg, 1.85 × 10^-2^ mmol) dissolved in chloroform (2.0 mL) was mixed with chloroform containing CeNP (10 mg). The mixture was stirred for 2 h at r.t. and then the solvents were completely evaporated by a rotary evaporator at 60 °C under vacuum for 30 min. Next, ddH_2_O (10 mL) was added and then dispersed by probe sonication for 5 min at 30% amplitude and full cycle. After filtration to remove the precipitate, the excess mPEG^2k^-DPSE was removed by dialysis against a 10 kDa molecular weight cut-off bag filter. Purified CeNP-PEG were stored in ddH_2_O at 4 °C.

**Fluorophore conjugated CeNP-PEG**

First, CeNP dispersed in chloroform were coated by amine-functionalized PEG-DSPE and DSPE-PEG co-polymer. The mPEG^2k^-DPSE (30 mg, 1.11 × 10^-2^ mmol) and NH_2_-PEG^2k^ -DSPE (20 mg, 0.72 × 10^-2^ mmol) dissolved in chloroform (2.0 mL) was mixed with chloroform containing CeNP (10 mg). The mixture was stirred for 2 h at r.t. and then the solvents were completely evaporated by a rotary evaporator at 60 °C under vacuum for 30 min. The ddH_2_O (4.5 mL) was added and then dispersed by probe sonication for 5 min at 30% amplitude and full cycle. After filtration to remove the precipitate, HEPES (0.5 mL, 1.0 M, pH 7.8) solution was dropwise added into the filtrate, and then 5 (6)-TAMRA SE (5.2 mg, 9.86 × 10^-3^ mmol) in DMSO (0.6 mL) was added to the CeNP-PEG-NH_2_ suspension. The solution was stirred overnight under N_2_ protection. The CeNP-PEG-Rho was purified by dialysis against a 10 kDa molecular weight cut-off bag filter and storage keep away from light at 4 °C.

**Characterization**

Transmission electron microscope (TEM) images and selected area electron diffraction (SAED) pattern were conducted using a JEOL 2100 (JEOL Ltd., Tokyo, Japan) TEM operated at 200 kV. The elemental compositional analysis of CeNP was carried out using an energy dispersive spectrometer (EDS) system attached to TEM. X-ray powder diffraction (XRD) patterns were recorded on a SmartLab XRD instrument (Rigaku Corporation, Tokyo, Japan). 2*θ* range was from 5 to 90° with Cu *Kα* radiation (λ = 0.154 nm), operated at 30 mA and 40 kV. Phase identification was performed by MDI JADE 5.0 software. The X-ray photoelectron spectra (XPS) were recorded using an ESCALAB 250Xi spectrometer (Thermo Scientific, MA, USA) equipped with monochromatized Al *Kα* radiation (hv = 1468.6 eV). All of the binding energies were referenced to the C1s peak at 284.8 eV. The hydrodynamic diameters and zeta potentials of CeNP-PEG were measured by dynamic light scattering (DLS) with a Zetasizer Nano-ZS system (Malvern Zetasizer Nano series, Malvern, UK) performed at 25 °C. The concentration of CeNP-PEG was determined by inductively coupled plasma-optical emission spectroscopy (ICP-OES, Agilent ICP-OES 720, Santa Clara, USA) after digestion using HNO_3_ (99%) and H_2_O_2_ (30%). The concentration of CeNP-PEG mentioned in the experiments indicates the ceria (Ce) concentration measured by ICP-OES.

**Electron spin resonance (ESR) measurement**

The pseudo-enzyme activity of CeNP-PEG to scavenge ROS such as •OH, O_2_^•-^ and H_2_O_2_ were measured using a Bruker Elexsys 580 spectrometer equipped with HS cavity (Billerica, USA). The •OH and O_2_^•-^ were trapped by 5,5-Dimethyl-1-pyrroline N-oxide (DMPO) (Dojindo, Kumamoto, Japan) forming the spin adduct DMPO/•OH and DMPO/•OOH, respectively. The •OH was generated using Fenton reaction by mixing 100 mM DMPO, 1.0 mM FeSO_4_, 1.0 mM H_2_O_2,_ and various concentrations of CeNP-PEG in ddH_2_O and the ESR spectrum of DMPO/•OH were recorded after 2 min. The O_2_^•-^ was generated by the hypoxanthine/xanthine oxidase (HYP/XOD) system from mixing 100 mM DMPO, 25 μM DTPA, 0.5 mM HYP, 0.1 U/mL XOD, and various concentration of CeNP-PEG in PBS (pH = 7.4) and the ESR spectrum of DMPO/•OOH were recorded after 2 min. ESR spin-label oximetry method was used to study the CAT-like mimetics activity of CeNP-PEG. 3-Carbamoyl-2,5-dihydro-2,2,5,5-tetramethyl-1H-pyrrol-1-yloxyl (CTPO) (J&K Scientific, Beijing, China) was used as a spin-label to real-timely study the O_2_ generation by CeNP-PEG mediated H_2_O_2_ decomposition. The ESR spectra of O_2_ was recorded after 2 min of mixing 0.1 mM CTPO, 25 mM H_2_O_2_ and various concentration of CeNP-PEG in PBS (pH = 7.4). The sample was deoxygenated with argon for 15 min prior to H_2_O_2_ added.

**Isolation and culture of bone marrow-derived macrophages**

The bone marrow-derived macrophages (BMDMs) were obtained as described previously.^[^[^3^](#_ENREF_3)^]^ Briefly, the femurs and tibia were separated from female C57BL/6 mice (6-8 weeks old), the epiphysis was cut and then used a syringe to flushed out bone marrow with ice-cold DMEM. Then lysis of red blood cells and filtered through a 70 μm mesh, bone marrow cells were collected by centrifugation. Bone marrow cells were resuspended and cultured in DMEM containing 20% FBS and 20 ng/mL M-CSF for 7 days before being used in the experiments.

**Cytotoxicity assay**

The isolated BMDMs were cultured in the 96-well plates (100 μL/well) and cultured in DMEM containing 20% FBS and 20 ng/mL M-CSF for 7 days. On day 7, BMDMs were exposed into completed DMEM media containing sequential concentrations of CeNP-PEG (0, 0.31, 0.63, 1.25, 2.50, 5.0, 7.5, 10.0, 15.0 or 20.0 μg/mL). After 48 h, the cell viability of BMDMs was detected by changing media with completed DMEM containing 10% CCK-8 reagent (100 μL/well) (meilunbio, Dalian, China). After incubation for another 2 h, the absorbance at 450 nm in each well was recorded on a microplate reader (Multiskan FC, Thermo, MA, USA). The viability of cells without any treatments was used as a control.

**Cellular uptake assay**

The isolated BMDMs were seeded in the 12-well plates (1 mL/well) and cultured in DMEM containing 20% FBS and 20 ng/mL M-CSF for 7 days. In a time-dependent experiment, BMDMs were incubated with 1.0 μg/mL CeNP- PEG-Rho contained DMEM for 15 min, 30 min, 1 h, 2 h, or 4 h, respectively. In a concentration-dependent experiment, BMDMs were exposed into completed DMEM media containing sequential concentrations of CeNP-PEG-Rho (0, 0.13, 0.25, 0.50, 1.0 or 2.0 μg/mL) for 2 h. After washed with PBS for 3 times, the cells were imaged by an inverted fluorescence microscope (DM 6B, Leica, Germany) or detected using flow cytometry (ACEA NovoCyte 3000, Agilent, USA) to confirm the cellular uptake of CeNP-PEG.

**Intracellular ROS scavenging efficiency of CeNP-PEG**

On day 7, BMDMs were incubated with various concentrations (0, 0.5 or 1.0 μg/mL) of CeNP-PEG-contained DMEM media for 2 h and then replaced with complete DMEM containing 10% FBS and 1.0 μg/mL LPS for 24 h. The BMDMs were then washed with PBS 3 times and incubated with 10 μM DCFH-DA for 20 min. After washed with PBS 3 times, the BMDMs were imaged used an inverted fluorescence microscope or collected for flow cytometry analysis.

**Modulating BMDMs phenotypic polarization efficiency of CeNP-PEG**

On day 7, isolated BMDMs were cultured with various concentration (0, 0.5, or 1.0 μg/mL) of CeNP-PEG-contained DMEM for 2 h and then changed to a fresh stimulation medium containing 10% FBS, 100 ng/mL LPS, and 20 ng/mL IFN-γ for 48 h except for the control group. The polarization of BMDMs was confirmed by detecting the expression of specific phenotypic markers using flow cytometry analysis. BMDMs were harvested by enzymolysis and centrifugation, then incubated with Fc block (Fcγ R III/II) and stained with the anti-mouse CD86-FITC antibody (Biolegend, San Diego, USA) according to the manufacturer’s instructions. The stained BMDMs were washed with PBS and then measured by a flow cytometer.

**Immunoblotting studies**

Proteins in BMDMs or colon tissues were extracted using immunoprecipitation assay (RIPA) lysis buffer or Nuclear and cytoplasmic protein extraction kit and the concentration were measured by BCA protein assay kit (Beyotime, P0009, Shanghai, China) according to manufacturer’s instructions. Equal amounts of proteins were processed by sodium dodecyl sulfate-polyacrylamide gel electrophoresis (SDS-PAGE) and transferred to polyvinylidene fluoride membranes (Millipore, IPVH00010, USA). The membranes were blocked with 5% nonfat dry milk in TBS containing 0.1% Tween 20 (TBST) for 1 h and were then incubated with following primary antibodies: rabbit anti-iNOS polyclonal antibody (absin, abs130136, 1:1000, Shanghai, China), rabbit anti-IL-1β polyclonal antibody (abcam, ab9722, 1:1000, Cambridge, UK), rabbit anti-IL-6 monoclonal antibody (CST, 12912S, 1:1000, MA, USA), rabbit anti-TNF-α antibody (CST, 3707S, 1:1000, MA, USA), rabbit anti-IFN-γ polyclonal antibody (absin, abs119966, 1:1000, Shanghai, China), rabbit anti-NLPR3 polyclonal antibody (Proteintech, 19771-1-AP, 1:500, Wuhan, China), rabbit anti-phospho NF-κB p65 polyclonal antibody (phospho S536, abcam, ab86299, 1:5000, Cambridge, UK), rabbit anti-iκB-α monoclonal antibody (abcam, ab32518, 1:5000, Cambridge, UK), rabbit anti-JAK2 monoclonal antibody (CST, 3230T1:1000, MA, USA), mouse anti-STAT3 monoclonal antibody (CST, 9139S, 1:1000, MA, USA), rabbit anti-phospho-STAT3 monoclonal antibody (phospho Tyr705, CST, 9145S, 1:2000, MA, USA), rabbit anti-GAPDH monoclonal antibody (Proteintech, 60004-1-1 g, 1:10000, Wuhan, China), rabbit anti-Histone H3 polyclonal antibody (Servicebio, GB11102, 1:500, Wuhan, China) at 4 °C overnight. After washed with TBST for 3 times, the membranes were treated with horseradish peroxidase (HRP)-labeled goat anti-rabbit IgG (H + L) (Beyotime, A0208, 1:1000, Shanghai, China) or HRP-labeled goat anti-mouse IgG (H + L) (Beyotime, A0216, 1:1000, Shanghai, China) for 1 h. Blots were developed using an ECL detection kit (share-bio, SB-WB012, Shanghai, China). The optical densities of the immune-active bands were analyzed using Quantity One software.

**DSS-induced IBD model**

All animal experiments were carried out in accordance with the approved guidelines of Guangzhou University of Chinese Medicine Institutional Animal Care and Use Committee. Healthy female C57BL/6 mice (6-8 weeks old) used for this study were housed in groups and acclimatized for 7 days before inclusion in experiments. Eighteen mice were randomly divided into three groups (n = 6): untreated group (control), DSS group, and DSS + CeNP-PEG group. Colitis was induced by 3% (w/v) DSS (molecular weight: 36-50 kDa, Yeasen, shanghai, China) supplemented in the drinking water for 7 days, followed by normal drinking water for 2 days. Control healthy mice were provided with normal drinking water only. To evaluate the effects of CeNP-PEG on DSS-induced colitis, 1.0 mg/kg of CeNP-PEG or PBS was intravenously injected into mice on days 3, 5, and 7.

***In vivo* therapeutic efficacy of drug-free nanozyme against IBD**

Mice body weight changes, visible stool consistency, and feces bleeding were individually monitored daily throughout the study. Each parameter was to assign a score according to the criteria previously proposed^[^[^4^](#_ENREF_4)^]^ and used to calculate an average daily disease activity index (DAI) (Table 1). All mice were euthanized on day 9, mice blood samples were collected and centrifuged to obtain serum for enzyme-linked immunosorbent assay (ELISA) analysis. Colons were isolated and lengths were measured. Part of the colons were dissociated to signal-cell suspensions for flow cytometry analysis, and part of the colons were used to extract RNA for the qRT-PCR experiment, or fixed in 4% paraformaldehyde and embedded in paraffin for histological examination. The dissected major organs including the liver, lung, spleen, kidney, and heart were weighed, then processed for histological detection.

Table 1 Scoring of disease activity index (DAI)

| Score | Weight loss (%) | Stool consistency | Feces bleeding |
| --- | --- | --- | --- |
| 0 | None | Normal | Negative hemoccult |
| 1 | 1-5 | Soft but still formed | Positive hemoccult |
| 2 | 6-10 | Very soft | Blood traces in stool visible |
| 3 | 11-15 | Diarrhea | Rectal bleeding |
| 4 | > 15 | Diarrhea | Rectal bleeding |

**Intestinal permeability**

The intestine mucosal epithelial barrier permeability was assessed using fluorescein isothiocyanate-dextran (FITC-dextran) as described elsewhere.^[^[^5^](#_ENREF_6)^]^ Briefly, after fasting for 24 h, mice (n = 4) were gavaged with FITC-dextran (Merck, average mol. wt. 3000-5000 Da) at a dose of 440 mg/kg on day 9. After 4 h, blood was collected and the serum FITC-dextran was quantified by fluorescence spectrophotometry using an excitation wavelength of 490 nm and an emission wavelength of 520 nm (Synergy H1, BioTek, USA).

**Dihydroethidium staining**

The colon samples were embedded in OCT and cut into serial 5-μm-thick sections. After incubated with 16 μM dihydroethidium (DHE) (Beyotime, Shanghai, China) in the dark at room temperature for 30 min, then washed with PBS 3 times and counterstained with DAPI. The fluorescence images were acquired with a Zeiss LSM 710 META confocal laser scanning microscope (Carl Zeiss, Jena, Germany) at emission wavelength 580 nm and excitation wavelength 480 nm, respectively. The mean fluorescence intensity of DHE was quantified by Image J software (US National Institutes of Health).

**Preparation of the lamina propria cell suspension**

Lamina propria (LP) cells were dissociated using a modification of a previously described method.^[^[^6^](#_ENREF_7)^]^ Briefly, Excised colons were placed in HBSS without calcium/magnesium (D-Hanks) and cleaned the contents using a syringe and cut longitudinally. After thrice vigorously vortex 15 s in D-Hanks, the colon tissues vigorously shaken in D-Hanks containing 2% FBS, 1 mM EDTA and 1mM DTT (both from Beyotime, Shanghai, China) for 40 min at 37 °C. After rinsed thrice with HBSS, cut the colons into pieces about 2 mm then digested at 37 °C for 1 h with 5% FBS, 1.0 mg/mL type IV collagenase (Worthington, Lakewood, New Jersey), and 0.3 mg/mL DNase I in RPMI 1640 medium (both from meilunbio, Dalian, China). The suspensions were passed through a 300-mesh strainer to remove the undigested tissues and then centrifugation to collect LP cells.

**Flow cytometry**

The LP pro-inflammatory macrophages were stained with the following monoclonal antibodies against the mouse: CD45-APC-Cy7 (BD Pharmingen, San Diego, USA), CD11b-FITC, and CD86-PE-Cy7 (Biolegend, San Diego, USA). CD45-APC-Cy7, CD3-PerCP-Cy5.5 (BD Pharmingen, San Diego, USA), CD4-FITC, IFN-γ-BV605 (Biolegend, San Diego, USA) were used to stained Th1 cells and CD45-APC-Cy7, CD3-PerCP-Cy5.5 (BD Pharmingen, San Diego, USA), CD4-FITC, IL-17A-BV421 (Biolegend, San Diego, USA) were used to stained Th17 cells. Cells and data were analyzed using a CytoFLEX flow cytometer (Beckman Coulter, Brea, USA).

**Cytokine levels**

The pro-inflammatory cytokine levels such as TNF-α, IL-6, IL-1β, and INF-γ in the cell culture medium or mice blood samples were measured by ELISA kit following the protocol from the manufacturer (Lianke Bio, Hangzhou, China). After adding the HRP substrates, the optical densities were analyzed at a wavelength of 450 nm by a multiskan spectrum microplate spectrophotometer (Synergy H1, BioTek, USA).

**Cytokine** **quantitative real-time polymerase chain reaction (****qRT-PCR)**

Total RNA from the BMDMs or mice colons was extracted with RNA isolater Total RNA Extraction Reagent and reverse transcription was performed using a HiScript^®^ III 1st Strand cDNA Synthesis Kit (+gDNA wiper) (both from Vazyme, Nanjing, China) according to the manufacturer’s protocol. Quantitative real-time PCR was performed using the Hieff^®^ qPCR SYBR green Gene Master Mix (No Rox) (Yeasen, Shanghai, China) on CFX96 (Bio-Rad). All qRT-PCR reactions were using an ABI 7500FAST Sequence Detector System (ABI, USA). The fold amplification of the target gene expression levels was calculated using the comparative method by normalization to the internal reference control GAPDH. The primers used for qRT-PCR were as the following: mouse TNF-α: Forward F, 5’-CCTGTAGCCCACGTCGTAGC-3’; reverse R, 5’-AGCAATGACTCCAAAGTAGACC-3’; mouse IL-6: F, 5’-ATCCAGTTGCCTTCTTGGGACTGA-3’; R, 5’- TGGCTAAGGACCAAGACCATCCAA-3’; mouse IL-1β: F, 5’- CTTCAGGCAGGCAGTATCACTC-3’; R, 5’- TGCAGTTGTCTAATGGGAACGT-3’; mouse INF-γ: F, 5’- AGCAACAGCAAGGCGAAA-3’; R, 5’-CTGGACCTGTGGGTTGTTGA-3’; and mouse GAPDH: F, 5’- GGAAGGTGAAGGTCGGAGT-3’; R, 5’- CCTGGAAGATGGTGATGGG-3’.

**Radiolabeling of [^18^F]-DPA-714**

[^18^F] fluoride was trapped on an anion-exchange cartridge (Waters SepPak Accell QMA cartridge carbonate), and subsequently eluted with a solution of K_2_CO_3_ (4.6 mg/mL) and Krypofix222 (10 mg/mL) in water/MeCN of ratio 1:6.5. Volatiles were removed at 120 °C with a moderate flow of nitrogen and anhydrous MeCN (3 × 1 mL) was added and evaporated for further azeotropic drying. 3 mg of DPA-714 in 1 mL anhydrous MeCN was added to the reaction vial and the reaction mixture was stirred at 120 °C for 15 min. The reaction was allowed to come to ambient temperature and then diluted with 1.5 equivalent of water. The crude solution was then passed through a solid-phase extraction cartridge (SPE, Sep-Pak® C18 Light) and the cartridge was rinsed with water (10 mL) to remove unreacted fluorion. The radiolabeled product was eluted from the cartridge with Methanol (1 mL) and further purified by semipreparative HPLC. The final product was collected and evaporated under vacuum and dispensed in saline containing 10% EtOH.

***In vivo* CT scans**

*In vivo* imaging experiments were performed with a microSPECT-CT (NanoScan®SC, Mediso, Hungary). PBS group, DSS group, and DSS + CeNP-PEG group (n ≥ 3) were performed imaging experiments. Mannite was orally administrated via gavage 30 min prior to CT scans to enhance the luminal visualization. First, non-enhanced CT was performed on three groups of mice with micro-CT, and then contrast media of iopamidol was intravenously administered at the dose of 250 mg I/kg body weight. After iopamidol administration. Mice were scanned at the venous stage (5-10 min post contrast media administration). CT images were acquired using the following parameters: slice thickness 100 μm, a field of view 100 mm, tube voltage 75 kV, tube current 100 μAl. Intestinal wall thickness was measured by segmentation of ascending, middle and descending colon, respectively. The attenuation values in Hounsfield units (HU) for corresponding colons were recorded from three different slices and averaged.

**PET/CT image acquisition**

PET/CT images were acquired on a Siemens INVEON PET/CT animal scanner. Total nine mice divided into three groups: PBS group, DSS group, and DSS + CeNP-PEG group (n = 3) were scanned. Mice were anesthetized using 3% isoflurane in pure oxygen and kept sedated throughout the whole acquisition. Each mouse was intravenously injected with 50 μCi. After intravenous administration of [^18^F] DPA-714, dynamic scanning was performed on each mouse in a time span of 60 min. [^18^F] DPA-714 images were binned into 27 frames. Frame 1-6 lasted 10 sec each. Frame 7-14 lasted 30 sec each. Frame 15-19 lasted 60 sec each. Frame 20-25 lasted 5 min each and frame 26-27 lasted for 10 min each. The energy discrimination was set at 350 and 650 KeV.

**Histological analysis**

The major organics and colon samples were cut into serial 5-μm-thick sections for hematoxylin and eosin (H&E) staining. IHC staining of CD11b and CD86 were performed using CD11b rat monoclonal antibody (Thermo Fisher, Scientific, MA1-80091, MA, USA) and CD86 recombinant rabbit monoclonal antibody (Thermo Fisher, Scientific, MA5-32078, MA, USA), respectively. Three independent slides were assessed per mouse by a blinded reviewer.

**Immunofluorescent staining**

The colon slides were fixed with 4% paraformaldehyde for 10 min at room temperature (r.t.), after washed with PBST 3 times, the slides were blocked by 3% fat-free milk in PBS for 1 h at r.t., then incubated with primary antibody including rat anti-human CD11b (Thermo Fisher Scientific, MA1-80091, 1:100, CA, USA) and rabbit anti-human CD86 (Thermo Fisher Scientific, MA5-32078, 1:100, CA, USA) at 4 °C for overnight. After rinsing with PBS for 3 times, the slides were incubated with secondary antibody including Alexa Fluor 647-conjugated goat anti-rabbit IgG (Yeasen, 33113ES60, 1:200, Shanghai, China) or Alexa Fluor 488-conjugated goat anti-rat IgG (Yeasen, 33316ES60, 1:200, Shanghai, China) for 1 h at r.t.. After staining the nuclear by DAPI, the slides were imaged under a Zeiss LSM 710 META confocal laser scanning microscope (Carl Zeiss, Jena, Germany).

**Statistical analyses**

All analyses were performed using GraphPad software. The results were expressed as mean ± SD (n ≥ 3). Statistical analysis was performed with one-way ANOVA. A *p* < 0.05 was considered statistically significant.

**References**

[1] C. K. Kim, T. Kim, I.-Y. Choi, M. Soh, D. Kim, Y.-J. Kim, H. Jang, H.-S. Yang, J. Y. Kim, H.-K. Park, S. P. Park, S. Park, T. Yu, B.-W. Yoon, S.-H. Lee, T. Hyeon, *Angew. Chem. Int. Ed.* **2012**, 51, 11039.

[2] F. Zeng, Y. Wu, X. Li, X. Ge, Q. Guo, X. Lou, Z. Cao, B. Hu, N. J. Long, Y. Mao, C. Li, *Angew. Chem. Int. Ed.* **2018**, 57, 5808.

[3] C. M. Lee, J. Hu, *Cell & Bioscience* **2013**, 3, 30.

[4] a) S. Wirtz, C. Neufert, B. Weigmann, M. F. Neurath, *Nat. Protoc.* **2007**, 2, 541; b) F. Xue, Y. Wang, Q. Zhang, S. Han, F. Zhang, T. Jin, C. Li, H. Hu, J. Zhang, *Nanoscale* **2018**, 10, 12364.

[5] Y. Zhao, Y. Yang, J. Zhang, R. Wang, B. Cheng, D. Kalambhe, Y. Wang, Z. Gu, D. Chen, B. Wang, Y. Huang, *Acta Pharm. Sin. B* **2020**, 10, 1966.

[6] Y. Lin, X. Yang, W. Yue, X. Xu, B. Li, L. Zou, R. He, *Cell. Mol. Immunol.* **2014**, 11, 355.

**Supplementary Figures**

**Table S1 The clinical information of mild and severe UC patients**

|  | Mild UC (n = 10) | Severe UC (n = 10) | *p* value |
| --- | --- | --- | --- |
| Male, n (%) | 6 (60.0) | 13 (72.2) |  |
| Mean age (yrs) | 36.1 | 33.9 |  |
| Mean disease duration (yrs) | 2.3 | 3.2 |  |
| Blood cytokines | | | |
| CRP (ng/L) | 4.76 ± 1.09 | 14.00 ± 3.77 | = 0.0018 |
| IL-6 (ng/L) | 43.99 ± 6.07 | 63.30 ± 9.23 | < 0.0001 |
| TNF-α (ng/L) | 32.41 ± 4.76 | 56.47 ± 7.68 | < 0.0001 |
| Baseline Treatment | | | |
| Mesalamine, n (%) | 5 (50) | 10 (55.6) |  |
| Steroids, n (%) | 0 (0) | 4 (22.2) |  |
| Anti-TNF, n (%) | 1 (10) | 6 (33.3) |  |
| Immunomodulator, n (%) | 1 (10) | 4 (22.2) |  |

yrs: years. CRP: C-reactive protein.


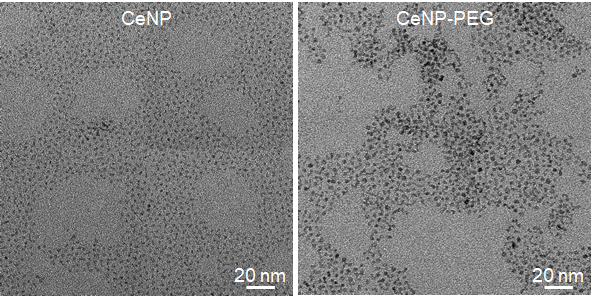


**Figure S1 Representative TEM images of CeNP and CeNP-PEG.** The CeNP prepared by reverse micelle method disperse as discrete, near-spherical structure, uniform shape and narrow size distribution. The morphology of the CeNP-PEG was similar to the CeNP. Scale bar = 20 nm.


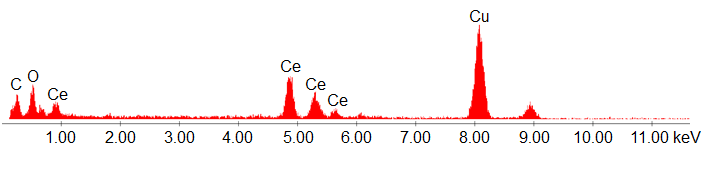


**Figrue S2 Chemical composition of the CeNP measured by energy dispersive spectrometer (EDS) analysis.** EDS spectra confirmed that the CeNP was composed of Ce and O elements. The Cu and C atom were come from carbon film.


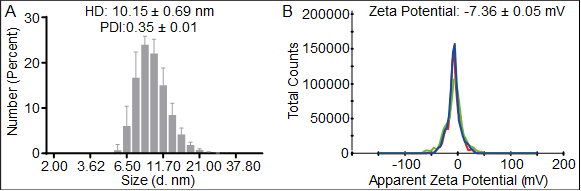


**Figure S3 The hydrodynamic diameter (HD) distribution and apparent zeta potential of CeNP-PEG.** (A) The hydrodynamic diameter distribution of CeNP-PEG determined by dynamic light scattering (DLS) in ddH_2_O. The mean diameter was 10.15 ± 0.69 nm, polymer dispersity index (PDI) was 0.35 ± 0.01 (n = 3). (B) The apparent zeta potential distribution of CeNP-PEG in ddH_2_O and the mean zeta potential was -7.36 ± 0.05 mV (n = 3).


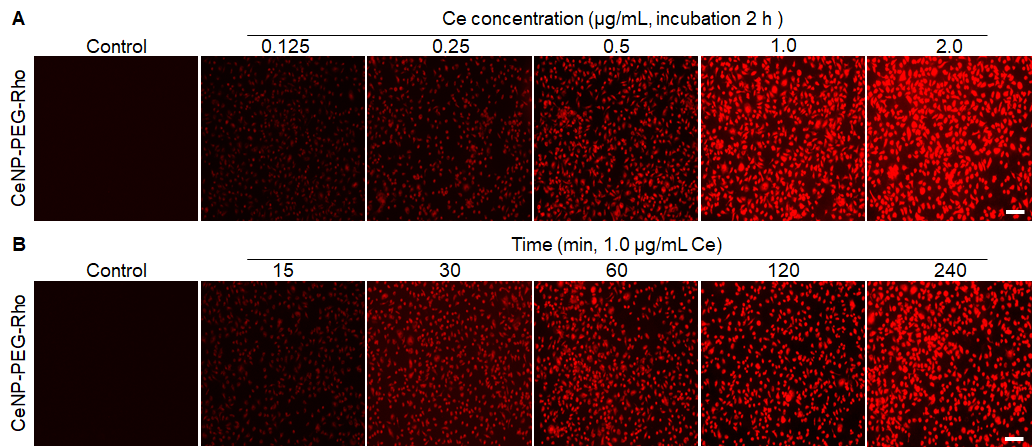


**Figure S4 Representative fluorescence images of rhodamine-conjugated CeNP-PEG (CeNP-PEG-Rho) in BMDMs.** (A) Concentration-dependent intracellular uptake of CeNP-PEG-Rho in BMDMs with a 2 h incubation time. (B) Time-dependent intracellular uptake of CeNP-PEG-Rho in BMDMs at 1.0 μg/mL CeNP-PEG. Scare bar = 100 μm.


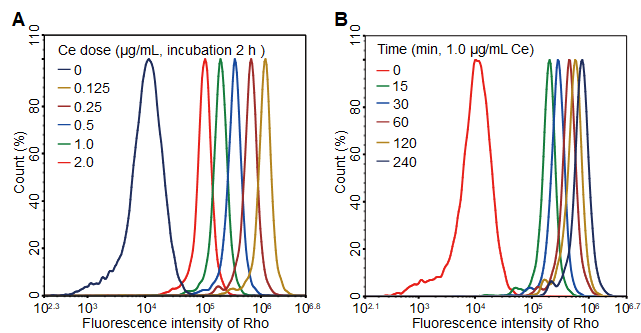


**Figure S5 Flow cytometry of CeNP-PEG-Rho in BMDMs.** (A) Concentration-dependent intracellular uptake of CeNP-PEG-Rho in BMDMs were measured by flow cytometry. (B) Time-dependent intracellular uptake of CeNP-PEG-Rho in BMDMs were measured by flow cytometry.


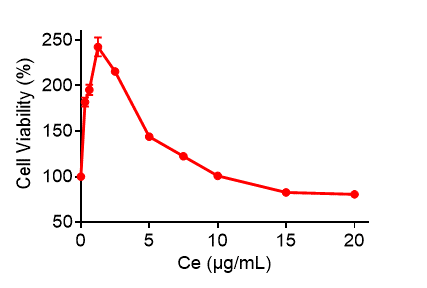


**Figure S6 Cytotoxicity of CeNP-PEG to BMDMs.** Viability of BMDMs post treatment of CeNP-PEG for 48 h with concentration ranged from 0 to 20 μg/mL. Cell viability was determined by CCK-8 assay (n = 3). Data are presented as the mean ± SD.


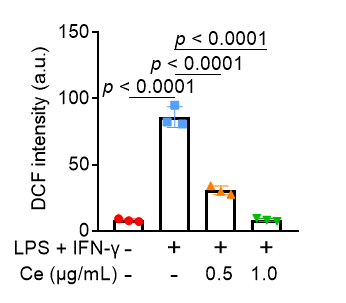


**Figure S7** Quantification of mean fluorescence intensity of intracellular ROS of figure 3E. The data are presented as the mean ± SD (n = 3). One-way ANOVA was used for statistical analysis. LPS: Lipopolysaccharide. DCF: 2’, 7’-Dichlorodihydrofluorescein. a.u.: relative units.


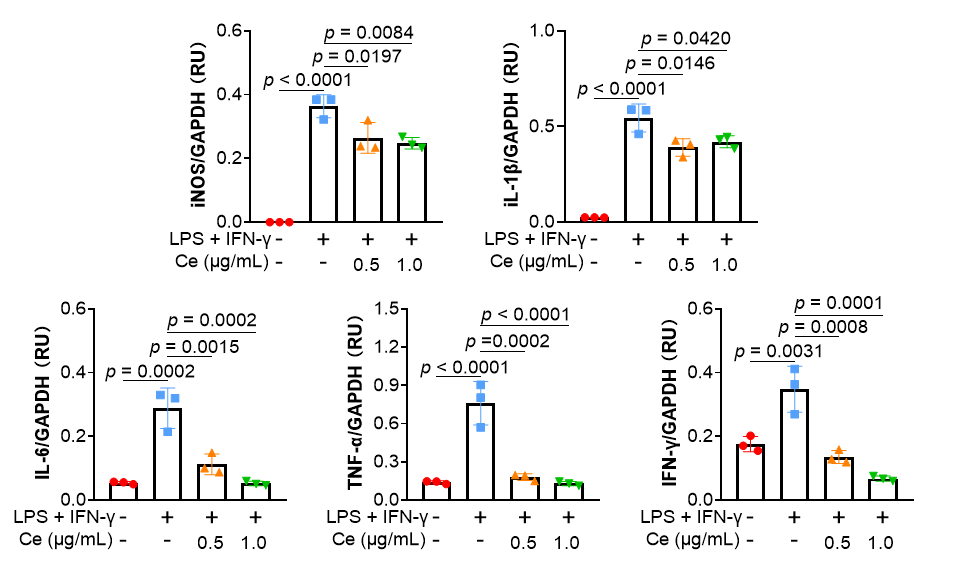


**Figure S8 Quantification of the immunities of the proinflammatory phenotype biomarkers in BMDMs demonstrated in figure 4C.** The data are shown as the mean ± SD (n = 3). One-way ANOVA was used for statistical analysis. RU: relative units.


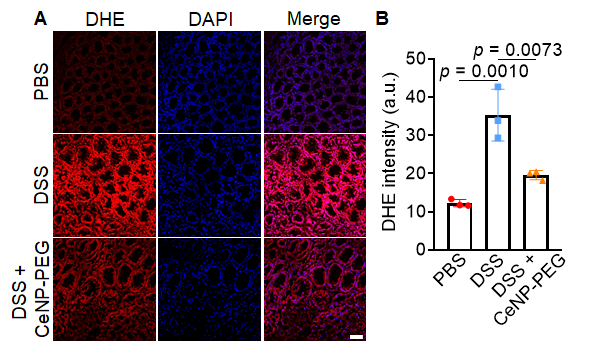


**Figure S9 Detection of colonic ROS by DHE staining.** (A) representative DHE fluorescence images of colonic tissues at day 9 post the treatment. Scale bar = 100 μm. (B) Statistical results of the mean fluorescence intensity of DHE in the respective groups. The data are presented as the mean ± SD (n = 3). One-way ANOVA was used for statistical analysis. DHE: dihydroethidium. a.u.: relative units.


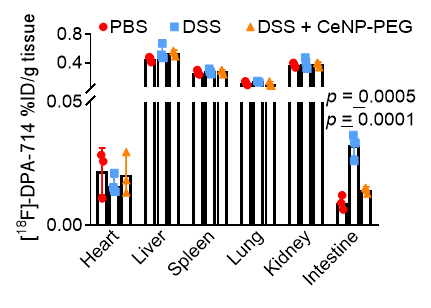


**Figure S10 The measurement dada of [^18^F]-DPA-714.** *Ex vivo* biodistribution of [^18^F]-DPA-714 in colitis models after different treatments. The data are presented as the mean ± SD (PBS and DSS + CeNP-PEG groups n = 3, DSS group n = 4). One-way ANOVA was used for statistical analysis.


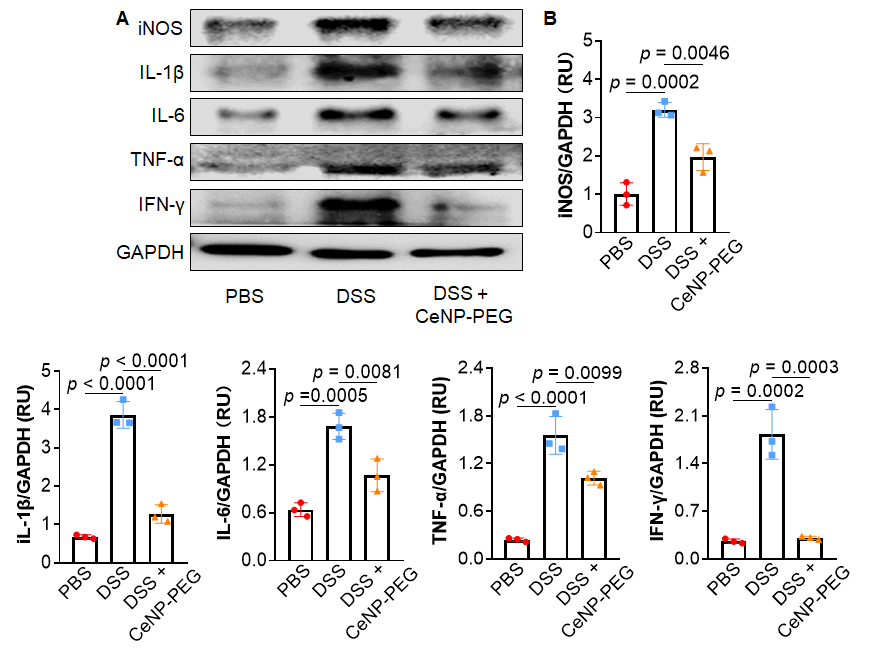


**Figure S11 Western** **blotting analysis of proinflammatory cytokines in the colonic tissues of colitis mice at day 9 post administration of CeNP-PEG.** (A) The protein level of pro-inflammatory phenotype biomarkers including iNOS, IL-1β, IL-6, TNF-α and INF-γ were measured by Western blotting. (B) Quantitative of protein immunities of figure A (n = 3). The data are shown as the mean ± SD. One-way ANOVA was used for statistical analysis. RU: relative units.


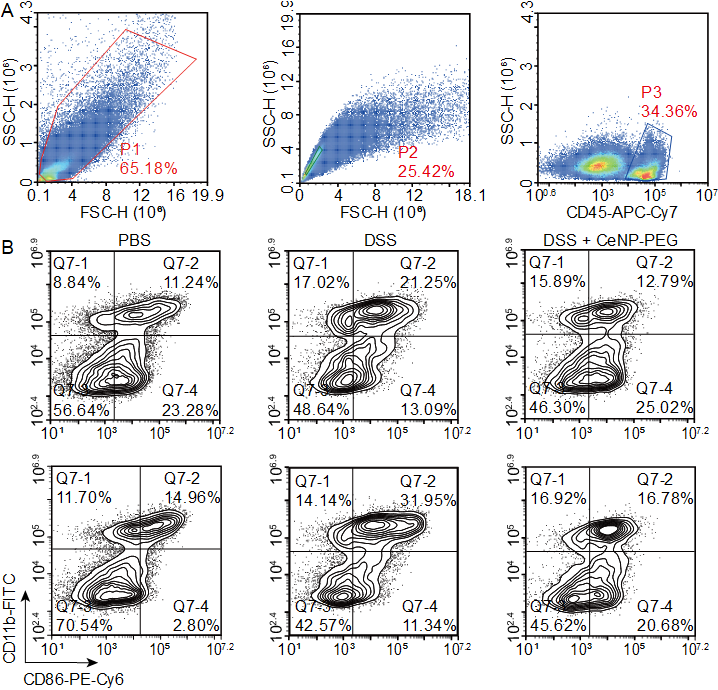


**Figure S12 Flow cytometry analysis of proinflammatory macrophages.** (A) The gating strategy to identify leukocytes. (B) The two other sets of flow cytometry analysis data of proinflammatory macrophages (CD11b^+^ CD86^+^) isolated from the colonic tissues at day 9.


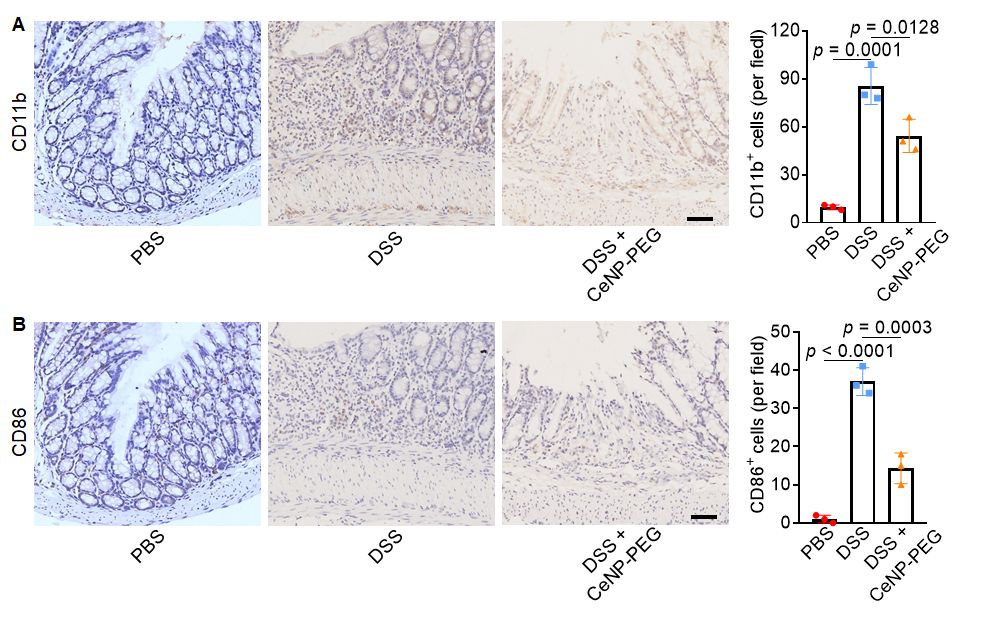


**Figure S13 Histological analysis of CeNP-PEG inhibited proinflammatory macrophages infiltration.** (A) Representative immunohistochemical staining of colonic sections for myeloid cells marker CD11b (left) and the numbers of CD11b^+^ cells (right) for each group at day 9 (n = 3). (B) Representative immunohistochemical staining of colonic sections for M1 macrophages marker CD86 (left) and the numbers of CD86^+^ cells (right) of each group at day 9 (n = 3). Scare bar = 50 μm. The data are shown as the mean ± SD. One-way ANOVA was used for statistical analysis.


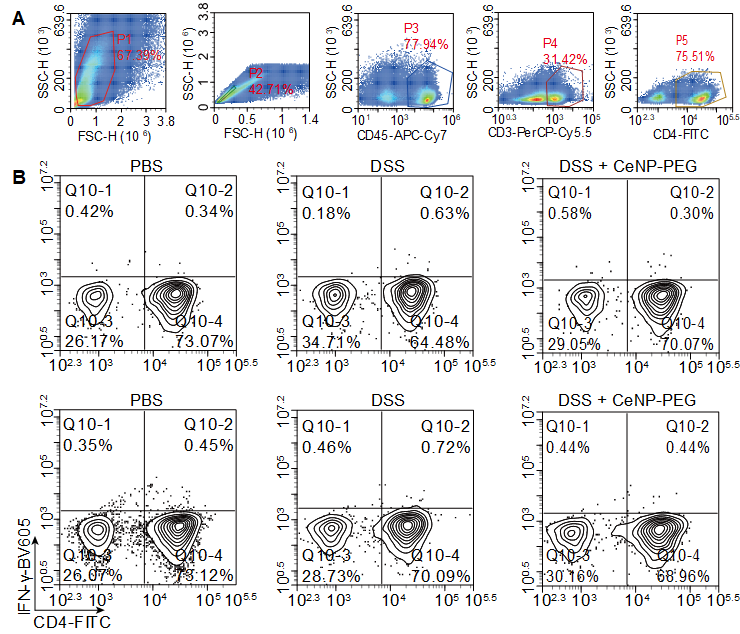


**Figure S14 Flow cytometry analysis of Th1 cells.** (A) The gating strategy to identify T cells. (B) The two other sets of flow cytometry analysis data of Th1 cells (CD4^+^ IFN-γ^+^) isolated from the colonic tissues at day 9.


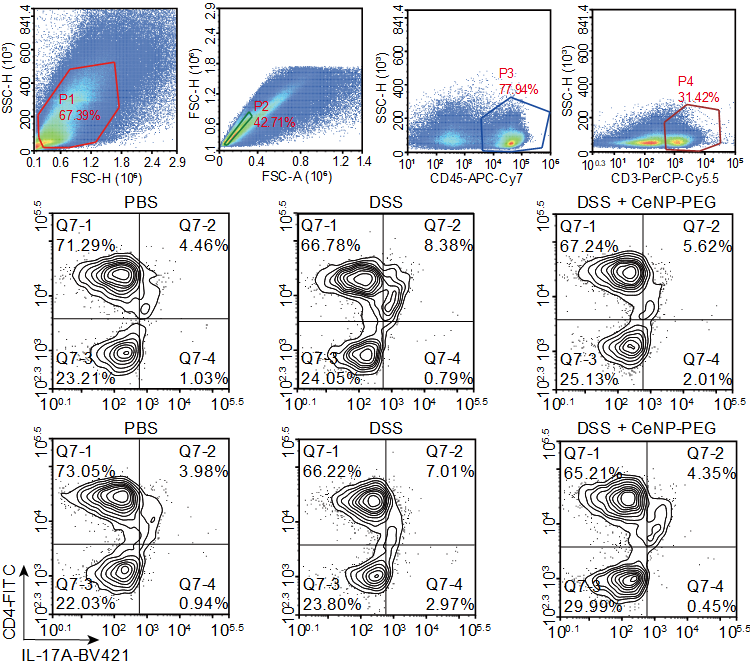


**Figure S15** **Flow cytometry analysis of Th17 cells.** (A) The gating strategy to identify T cells. (B) The two other sets of flow cytometry analysis data of Th17 cells (CD4^+^ IL-17A^+^) isolated from the colonic tissues at day 9.


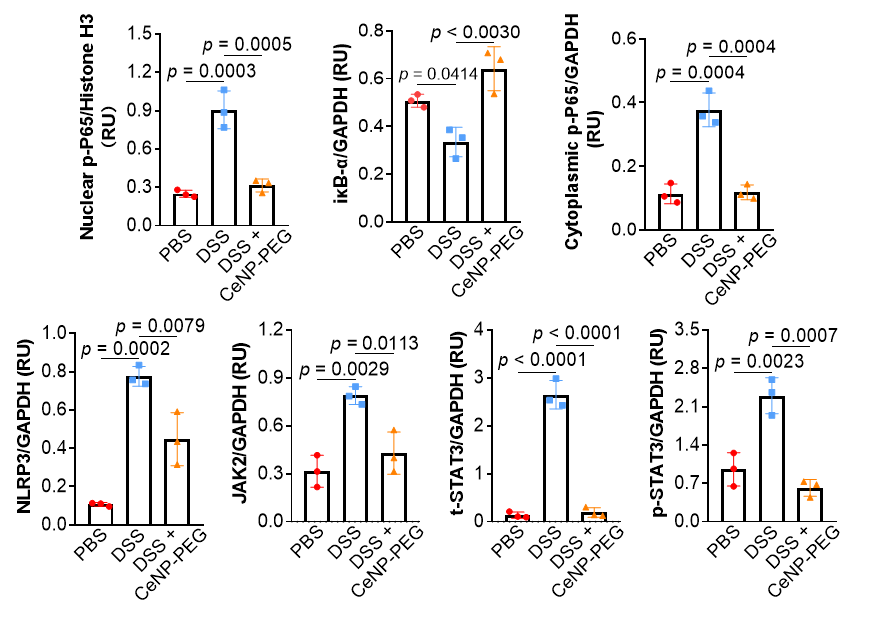


**Figure S16 Quantification of the immunities of the key proteins in the NF-κB and STAT3 signaling pathway demonstrated in figure 7G.** The data are shown as the mean ± SD (n = 3). One-way ANOVA was used for statistical analysis. RU: relative units.


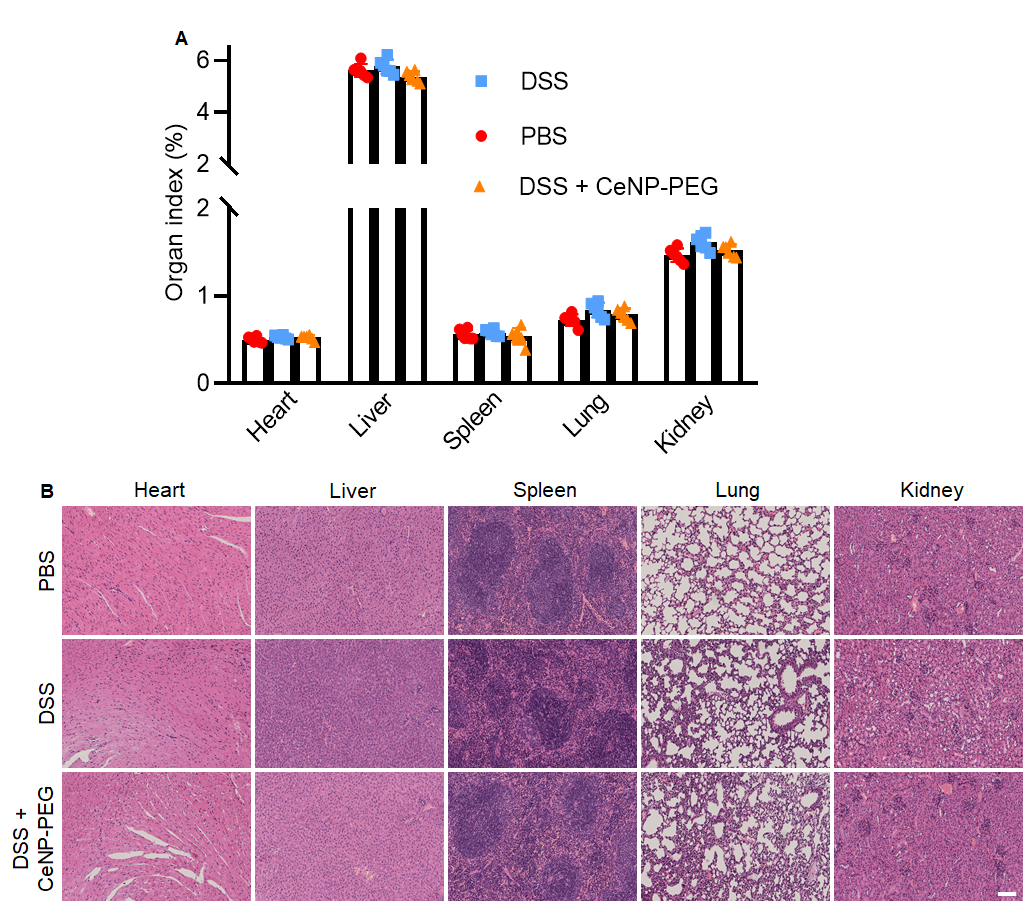


**Figure S17 Preliminary biosafety evaluation of CeNP-PEG.** (A) The organ indexes of different groups were evaluated at day 9 and there was no significant difference among the groups (n = 6). (B) Representative images of H&E-stained sections of major organs from each group and no detectable pathological changes were observed. Scale bar = 100 μm. The data are shown as the mean ± SD.
